# Targefrin: A Potent Agent Targeting the Ligand Binding Domain of EphA2

**DOI:** 10.1021/acs.jmedchem.2c01391

**Published:** 2022-11-04

**Authors:** Carlo Baggio, Parima Udompholkul, Luca Gambini, Maurizio Pellecchia

**Affiliations:** Division of Biomedical Sciences, School of Medicine, University of California Riverside, 900 University Avenue, Riverside, California 92521, United States

## Abstract

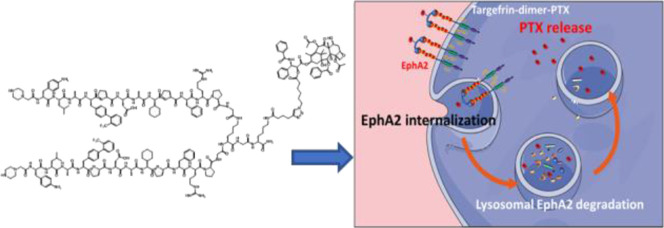

Overexpression of the receptor tyrosine kinase EphA2 is invariably associated with poor prognosis and development of aggressive metastatic cancers. Guided by our recently solved X-ray structure of the complex between an agonistic peptide and EphA2-LBD, we report on a novel agent, targefrin, that binds to EphA2-LBD with a 21 nM dissociation constant by isothermal titration calorimetry and presents an IC_50_ value of 10.8 nM in a biochemical assay. In cell-based assays, a dimeric version of the agent is as effective as the natural dimeric ligands (ephrinA1-Fc) in inducing cellular receptor internalization and degradation in several pancreatic cancer cell lines. When conjugated with chemotherapy, the agents can effectively deliver paclitaxel to pancreatic cancers in a mouse xenograft study. Given the pivotal role of EphA2 in tumor progression, we are confident that the agents reported could be further developed into innovative EphA2-targeting therapeutics.

## Introduction

**Figure 1:**
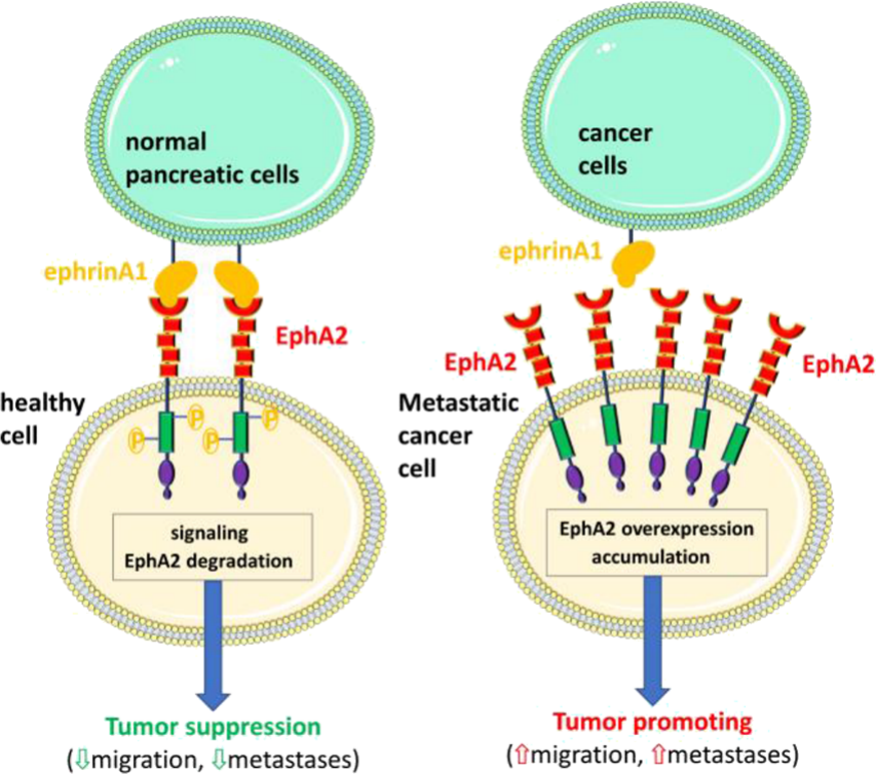
Schematic representation of pro-oncogenic unbound EphA2 in cancer cells.

The receptor tyrosine kinase EphA2 in its ephrin-bound form functions as a tumor suppressor, preventing cancer cell migration, tumor growth, and angiogenesis. On the contrary, when the receptor is in its unbound state, such as when it is aberrantly overexpressed, it confers cancer cells pro-oncogenic traits inducing metastatic behavior in several solid tumors (Figure 1) including pancreatic cancer,^2−4^ prostate cancer,^5−7^ breast cancer,^8−10^ esophageal cancer,^11^^,^^12^ melanoma,^13^ urinary bladder,^14^ brain cancer,^15−17^ lung cancer,^18^ ovarian cancer,^19^ stomach cancer,^20^ and some types of leukemia.^21−24^ Hence, due to its role as the tumor suppressor, targeting EphA2 is being targeted for the development of various possible therapeutic strategies, including targeting its intracellular kinase domain^25−28^ or its ligand binding domain (LBD).^29,30^ While the unbound EphA2 receptor functions as the potent oncogene, its tumorigenic effect could be suppressed, and perhaps reverted, by synthetic agents that mimic its ligand, the membrane-anchored ephrinA1.^31^

In cellular assays, when a chimeric protein consisting of ephrinA1 and the Fc region of an antibody, ephrinA1-Fc, engages with the EphA2 LBD, it causes receptor dimerization, followed by clustering and internalization that results in the degradation of the receptor via a lysosomal pathway.^32^ Therefore, because ephrinA1-Fc could in principle revert pro-oncogenic EphA2 into a tumor suppressor, the design of potent and effective ephrinA1-Fc mimetics holds great potential for the development of novel anti-metastatic therapeutics. Because such agents would also cause receptor internalization, these could be additionally deployed as carrying molecules for selective targeted delivery of chemotherapy to EphA2-expressing cancers. In this regard, we have recently developed a novel EphA2 dimeric agonistic peptide mimetic that, similar to ephrinA1-Fc, could suppress tumor metastases in an orthotropic model of prostate cancer^33^ and suppresses cell migration in pancreatic cancer cell lines.^34^ When similar earlier agents were conjugated with the chemotherapeutic agents gemcitabine^35^ or paclitaxel,^8,36,37^ these resulted in effective delivery of their cargo to EphA2-expressing tumors, including pancreatic cancer,^35^ prostate cancer,^36,37^ breast cancer,^8,38^ and melanoma.^38^ More recently, we solved the X-ray structure for the first time of an agonistic ephrin peptide mimetic in complex with EphA2-LBD.^1^ Leveraging on previous structure–activity relationship studies on previous peptide binders from our laboratory^1,37,38^ and the high-resolution X-ray structure,^1^ we sought here to further derive agents that could approach the affinity and activity of ephrinA1-Fc in targeting EphA2-LBD. Our studies culminated in the identification of a novel agent with low nanomolar affinity for EphA2-LBD, which therefore presents a comparable affinity for the receptor as ephrinA1. In cellular assays, a dimeric version of our most potent agent, we termed targefrin, induces receptor degradation at nanomolar concentrations, similar to the effect of ephrinA1-Fc, as assessed by western blot analysis in pancreatic cancer cell lines BxPC3, PANC-1, and MIA PaCa2, representing KRAS wild-type (BxPC3) and KRAS-mutant (PANC-1 and MIA PaCa2) tumors. In phenotypic assays, the agents are also effective in suppressing cell migration in the BxPC3 pancreatic cancer cell line. When conjugated with paclitaxel, the agent is effective in suppressing tumor growth in a MIA PaCa2 xenograft model of pancreatic cancer. The extraordinary affinity of targefrin for the LBD of EphA2 makes this agent an unprecedented pharmacological tool to study this receptor tyrosine kinase and for the development of novel therapeutics and/or targeted delivery strategies.

## Results

### Design, Synthesis, and Characterization of Targefrin

**Figure 2:**
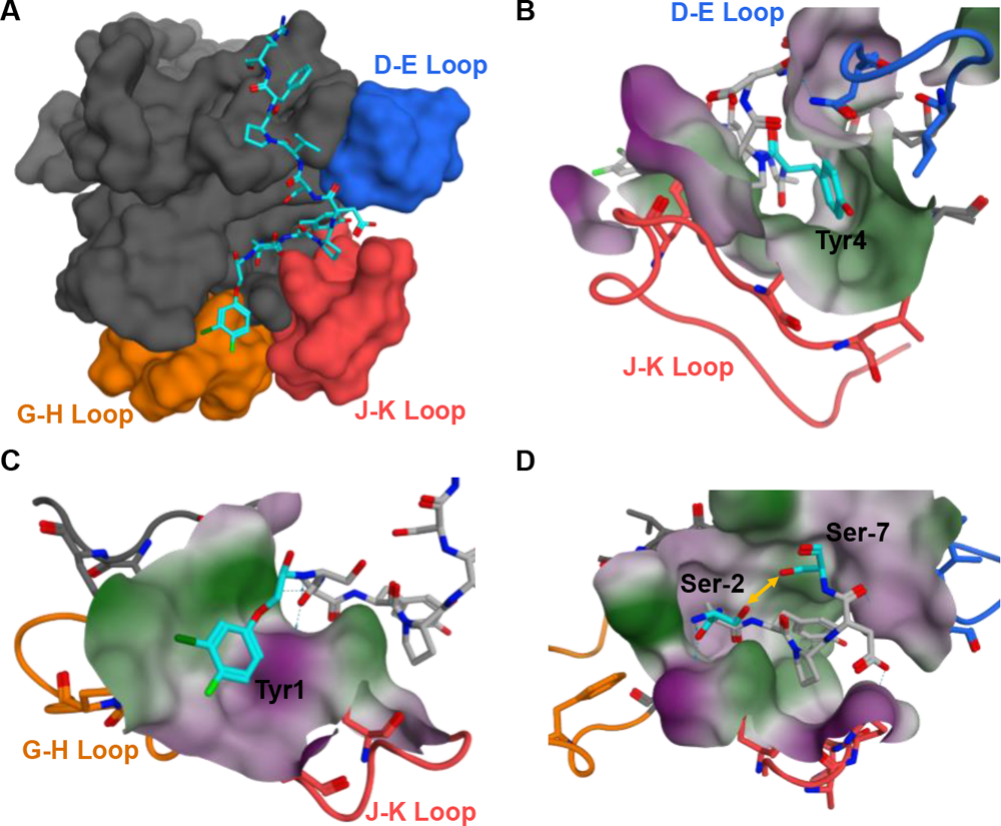
Structural details relative to our optimization process. (A) Structure of EphA2-LBD in complex with an earlier agent developed in our laboratory.^1^ The surface of the receptor is indicated in dark gray, with the D–E, G–H, and J–K loops depicted in blue, orange, and red, respectively. (B) Detail of the tyrosine residue in position 4 of the EphA2 binding agent that protrudes into a large hydrophobic pocket located between the D–E and J–K loops. (C) Detail of position 1 of the EphA2 binding agent that substitutes tyrosine 1 and N-terminal amide of the YSA peptide, located between the G–H and J–K loops. (D) Detail of the pair of serine residues in the EphA2 binding agent forming an intramolecular hydrogen bond (yellow arrow), constraining the peptide in a close conformation in its bound form. Surfaces in (B–D) are depicted in green for lipophilic, white for neutral, and purple for hydrophilic.

In order to rapidly and iteratively characterize the binding properties of novel EphA2 binding ligands (Table 1), we performed isothermal titration calorimetry (ITC) binding measurements using recombinant EphA2 LBD. Analysis of the structure of one of our earlier agents in complex with EphA2-LBD from our laboratory (PDB ID 6B9L) revealed possible avenues for optimizations (Figure 2).^1^ Using our previously identified starting ligand of sequence YSAYPDSVPFRP (*K*_d_ 1230 nM, ITC; Table 1, compound **1**), that merged the sequences of the phage display-derived YSA peptide^39^ with the sequences of natural ephrin ligands,^1^ we started exploring possible optimization strategies (Figure 2). First, we probed substitutions that could protrude into a larger hydrophobic pocket located in proximity of the Tyr 4 of the peptide (Figure 2A,B, Table 1). Here, replacement of the Tyr residue in position 4 with bulkier aromatic groups enhanced the affinity significantly (Table 1). Hence, subsequently, fixing a phenyl-Phe in position 4 of the peptide, we explored modifications at other positions.

**Table 1:**
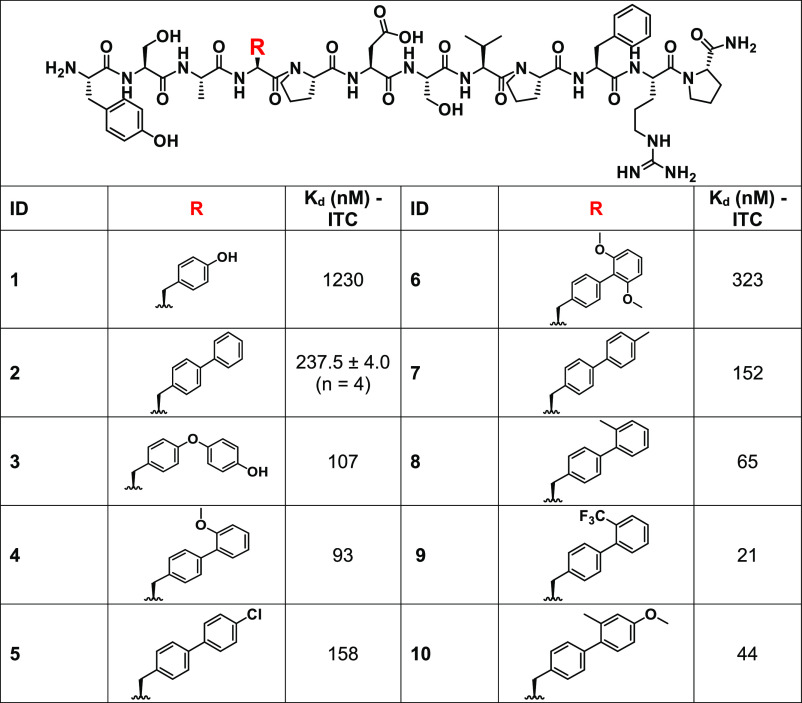
Structure–Activity Relationship Studies in Position 4 for the Reported EphA2 Binding Agents^a^

a Chemical structures and dissociation constants are reported. *K*_d_ values were obtained by reverse ITC measurements.

These included modifications of the N-terminal amide (Figure 2A,C, Table 2) as we had previously demonstrated that this position is not only susceptible to amino-peptidases in plasma but also very important for ligand recognition.^1,37,38^ Indeed, replacement of the amino group with a piperazine or a morpholino increased the binding affinity for EphA2-LBD (Table 2). Moreover, we explored additional modifications along the sequence based on our previous SAR studies,^1^ including modification of the Tyr residue in position 1 and the pair of Ser residues (Table 2). We found that Tyr 1 could be replaced by a variety of substituents, thus eliminating the potential pharmacological liability represented by the phenolic hydroxyl group (Table 2). In its bound state, the peptide assumes a closed conformation with the two Ser residues forming intramolecular hydrogen bonding in the initial peptide (Figure 2A,D), and we had previously replaced these hydrophilic interactions with either a disulfide bridge or hydrophobic interactions, both resulting in increased binding affinity for EphA2-LBD.^1^ Hence, we further explored additional modifications of this pair of amino acids and assessed their influence on the binding affinity for EphA2-LBD via ITC measurements (Table 2).

**Table 2:**
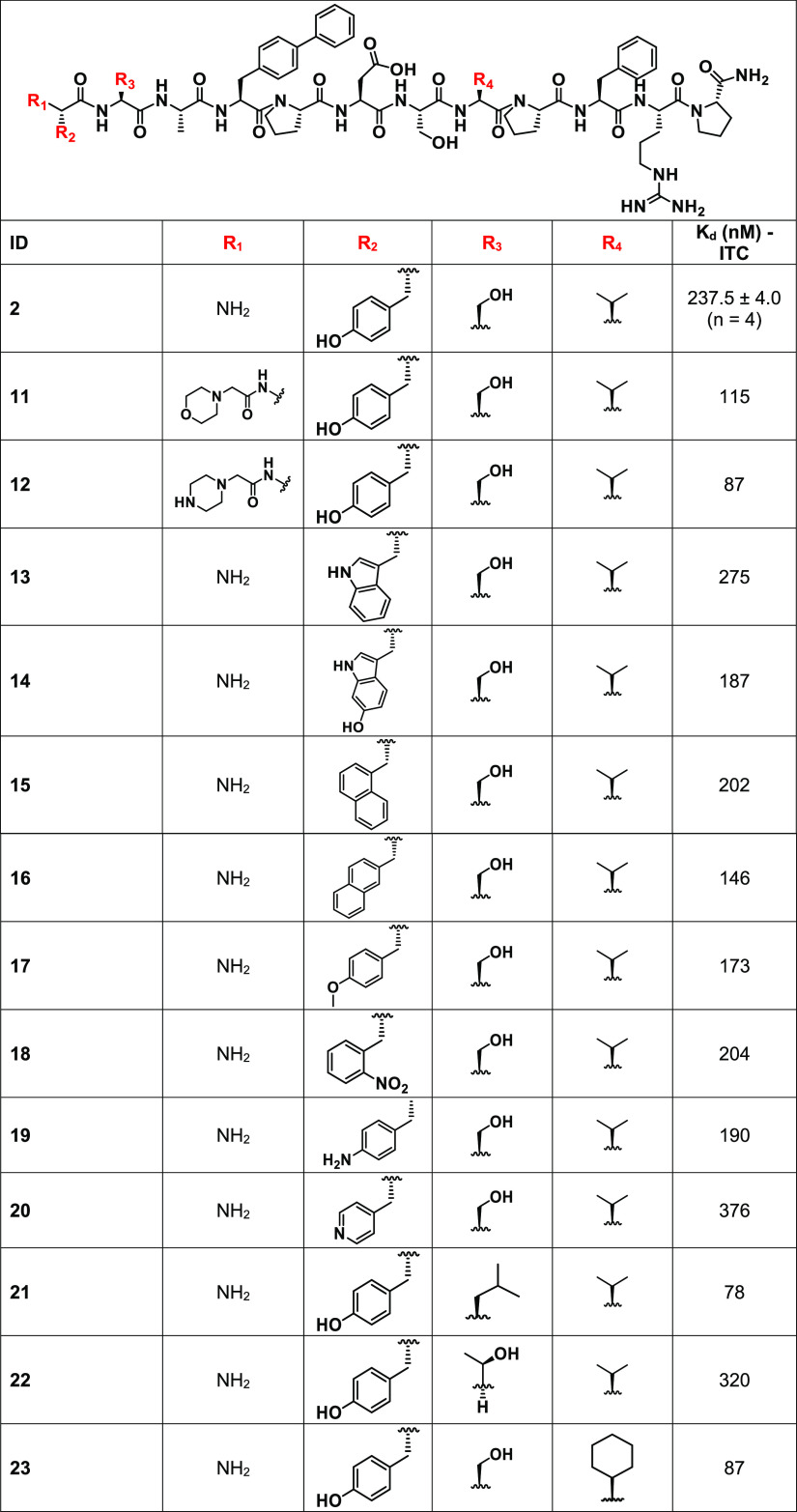
Chemical Structures and Dissociation Constants for EphA2 Binding Agents^a^

a *K*_d_ values were obtained by reverse ITC measurements.

Finally, a set of peptides were synthesized that contained optimal substituents from the agents reported in Tables 1 and 2, resulting in the final agents listed in Table 3. For these compounds, we also assessed their binding properties using an orthogonal biochemical displacement assay based on the DELFIA platform, as we described previously.^1^

**Table 3:**
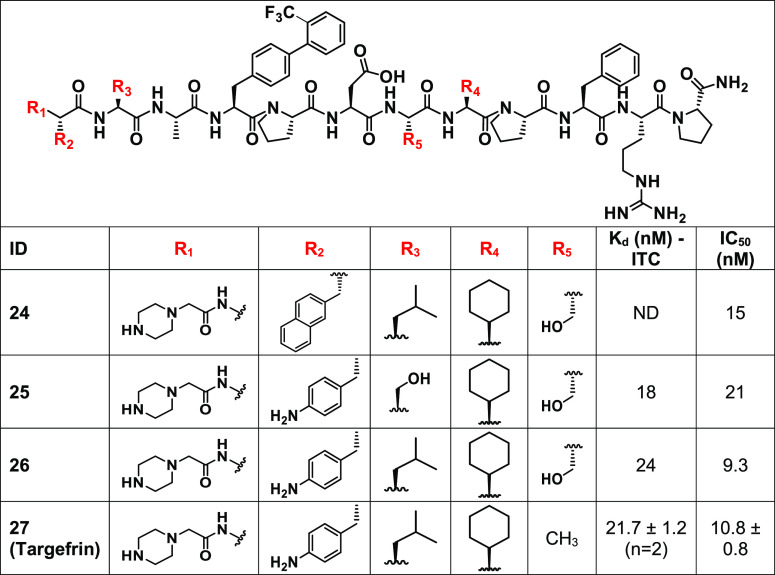
Chemical Structures and Dissociation Constants for EphA2 Binding Agents Containing Optimal Substituents from Previous SAR^a^

a For each compound, we reported the *K*_d_ value obtained by ITC and the IC_50_ value resulted from DELFIA displacement measurements.

**Figure 3:**
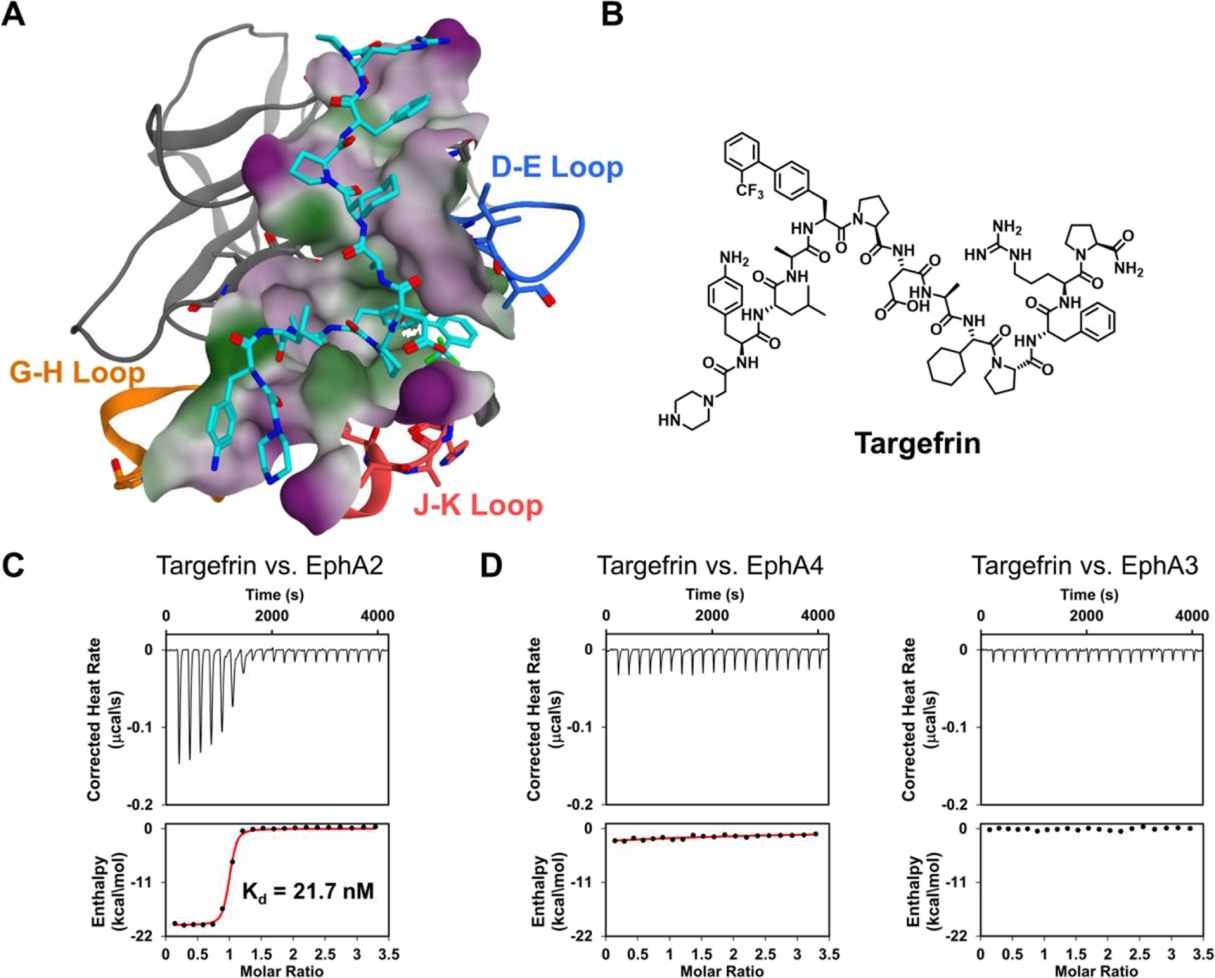
Modeling and binding data for targefrin. (A) Molecular model of targefrin in complex with EphA2-LBD based on the X-ray structure of the complex with one of our earlier peptides (PDB ID 6B9L). The surface of the binding pocket is colored according to a lipophilic potential (green for lipophilic, white for neutral, and purple for hydrophilic). The D–E, G–H, and J–K loops are colored in blue, orange, and red, respectively. (B) Chemical structure of targefrin. (C) ITC curve for the binding between targefrin and EphA2-LBD (*K*_d_ = 21.7 ± 1.2 nM; ΔH = −20.2 ± 0.4 kcal/mol; −*T*Δ*S* = 9.7 ± 0.4 kcal/mol). (D) ITC curves for binding between targefrin and EphA4-LBD and EphA3-LBD, the two Eph receptors with the greatest similarities to EphA2. The data indicated no appreciable binding under these experimental conditions.

These studies culminated in the selection of agent **27**, we term here targefrin, with an IC_50_ value of 10.8 nM for EphA2-LBD (Table 3). Figure 3 reports a molecular model of targefrin in complex with EphA2-LBD based on the X-ray structure of the complex with one of our earlier peptide mimetics (PDB ID 6B9L).^1^ To obtain a preliminary yet significant snapshot on the selectivity of targefrin for EphA2-LBD, compared to other members of this protein family, we tested it against the LBDs of EphA3 and EphA4 that are the two Eph receptors with the greatest similarities to EphA2 (58% identity with EphA3-LBD and 57% identity with EphA4-LBD). The ligand became inactive against both domains when tested under similar experimental conditions (Figure 3).

**Figure 4:**
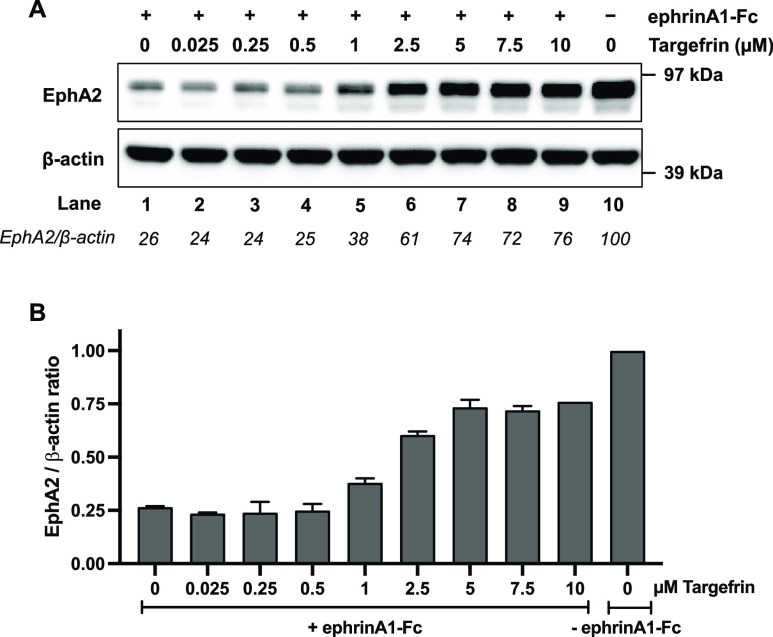
Targefrin functions as an antagonist. (A) Western blot of BxPC3 cells which were starved for 1 h and pre-treated with various concentrations of targefrin for 20 min, followed by a combination treatment with 2 μg/mL ephrinA1-Fc for 3 h. (B) Quantification of the EphA2 level. EphA2/β-actin ratios were normalized by designating the EphA2 expression from DMSO without ephrinA1-Fc condition as 1. EC_50_ value was calculated to be 1.6 ± 0.1 μM and was presented as mean ± standard error (SE) of two independent experiments.

We previously reported that monomeric peptides elicit agonistic activities only at very high concentrations and act practically as antagonists at physiologically attainable concentrations.^8,34,40^ In agreement with its high affinity for EphA2, pre-treatment of the BxPC3 pancreatic cancer cells with targefrin effectively antagonized EphA2 degradation induced by the potent ephrinA1-Fc ligand, with an approximate EC_50_ ∼ 1.6 μM under these experimental conditions (Figure 4).

**Figure 5:**
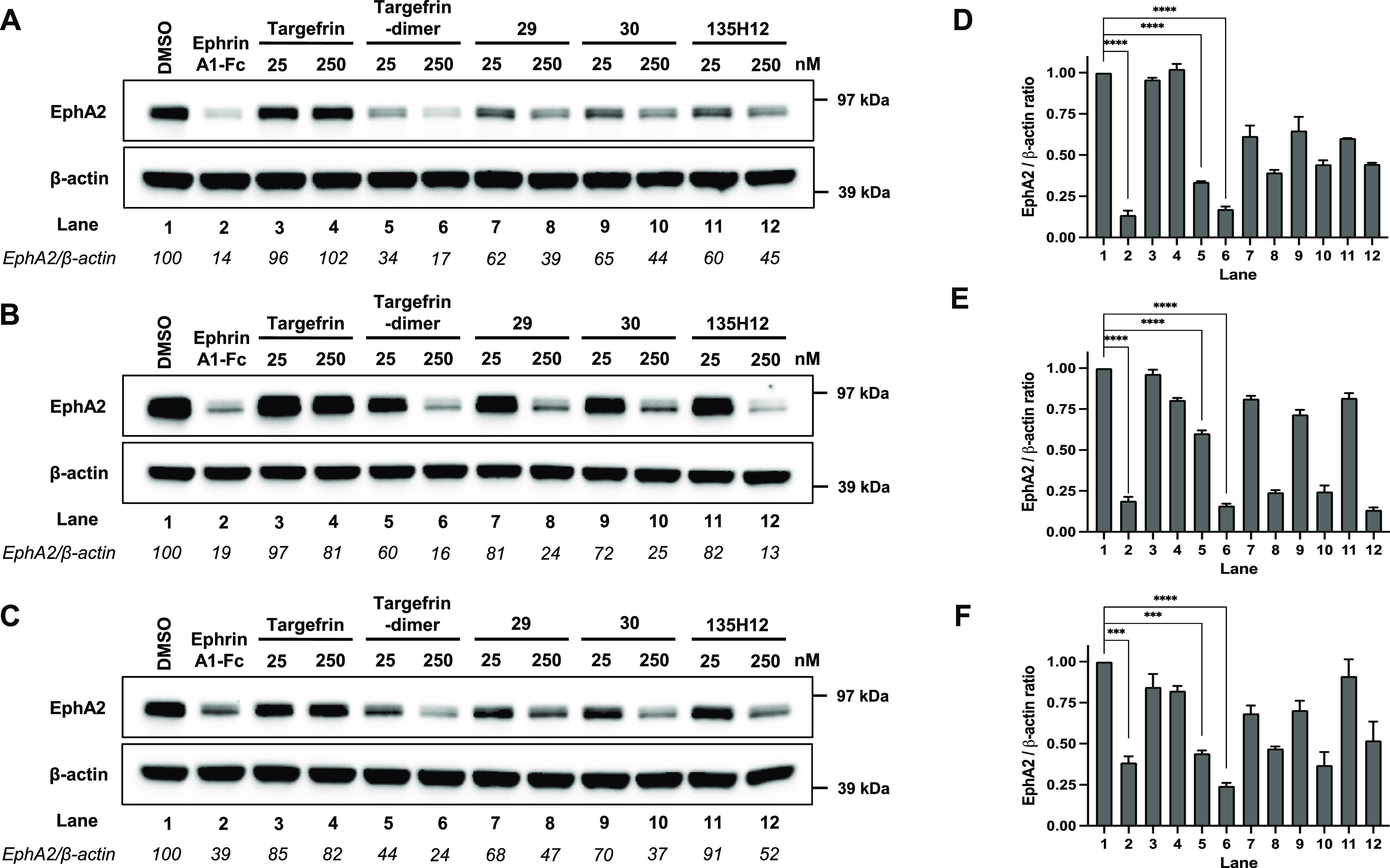
Targefrin-dimer and its variations cause EphA2 degradation at nanomolar concentrations in pancreatic cancer cell lines. (A–C) Western blot images of BxPC3, PANC-1, and MIA PaCa-2 cells, respectively, in which cells were starved for 1 h and treated with 2 μg/mL ephrinA1-Fc or the indicated doses of targefrin and targefrin-dimer and its variations with different linkers (Table 4) for 3 h. Previous dimeric agent 135H12^1^ is also shown as reference. (D–F) Densitometry analyses for the data shown in (A–C), respectively. EphA2/β-actin ratios were normalized by designating the EphA2 expression from the DMSO control condition as 100% for (A–C) or 1 for (D–F). ***p < 0.001, ****p < 0.0001, as determined by a one-way analysis of variance using Dunnett’s post-test analysis.

In agreement, the agent alone did not induce appreciable EphA2 degradation in BxPC3 pancreatic cancer cells (Figure 5).

However, while the monomeric peptides act as antagonists, we and others previously reported that, similar to ephrinA1, dimerization of EphA2-targeting agents resulted in compounds with dramatically increased agonistic activity in cell.^1,8,33,34,41,42^ This is due presumably to the fact that enhancing dimerization facilitates subsequent receptor clustering and internalization.^8,42^ Therefore, we prepared dimeric versions of targefrin (Table 4), using a Lys residue as a dimerization linker, spaced by Gly, β-Ala, or γ-amino butyric acid at the C-terminus of targefrin (Table 4). Table 4 also reports our previously identified dimeric agent 135H12.^1^ Based on our previous experience with such dimeric agents, we did not expect that our binding or displacement assays against the isolated EphA2-LBD in solution would reveal increased affinities. However, we did expect these dimeric agents to possess markedly increased receptor activation activity in cellular assays, as discussed below.

**Table 4:**
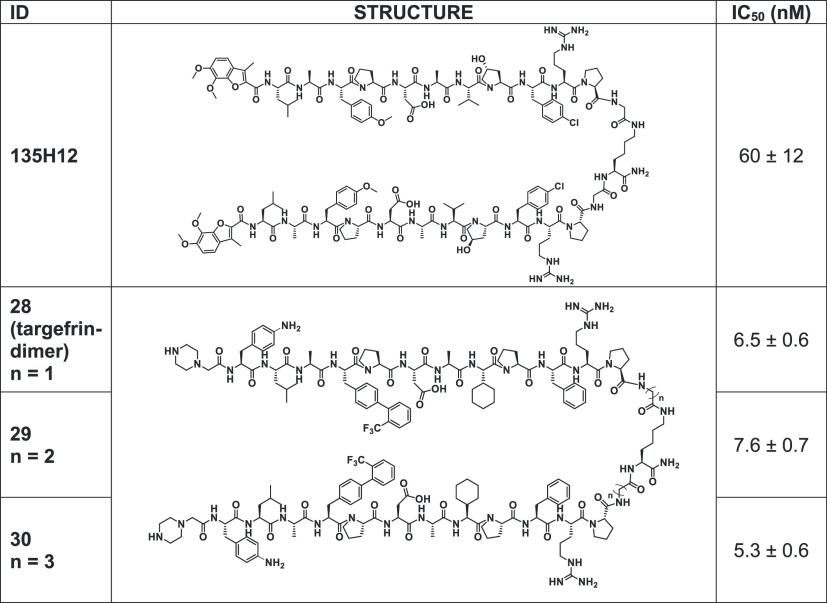
Chemical Structures of the Dimeric EphA2 Binding Agents^a^

a IC_50_ values were obtained by replicate DELFIA measurements.

### Targefrin-Dimer and Targefrin-Drug Conjugates

A property of agonistic agents that is of interest to our applications is that they induce EphA2 receptor internalization via a lysosomal pathway that causes its degradation. Hence, potent agonistic agents could induce EphA2 degradation, therefore eliminating its pro-oncogenic effects. We do not expect the EphA2 internalization induced by agonistic agents would affect cell proliferation, as we note in Supporting Information Figure S12. However, due to the lysosomal internalization event, EphA2 agonistic agents could be used for targeted delivery of cytotoxic chemotherapy by synthesizing suitable peptide-drug conjugates (PDCs). Hence, to assess the EphA2 internalization and degradation properties of our agents, we tested them in a variety of pancreatic cancer cell lines, side by side with dimerized ephrinA1-Fc as positive control. As reported above, when tested at nanomolar concentrations, the monomeric version of targefrin is not active in causing EphA2 degradation, in agreement with our previous observations that monomeric peptides are agonistic only at higher micromolar concentrations. This appears to be the case for all three cell lines tested, BxPC3, PANC-1, and MIA PaCa2 (Figure 5).

However, dimeric versions of targefrin displayed a markedly increased receptor activation especially with the dimer having the Gly-Lys linker (Table 4) causing receptor degradation at sub-micromolar concentrations for all the pancreatic cancer cell lines tested (Figure 5). Moreover, our newer agents are markedly more effective than our previously reported dimeric agent 135H12 (Table 4, Figure 5).^1^

**Figure 6:**
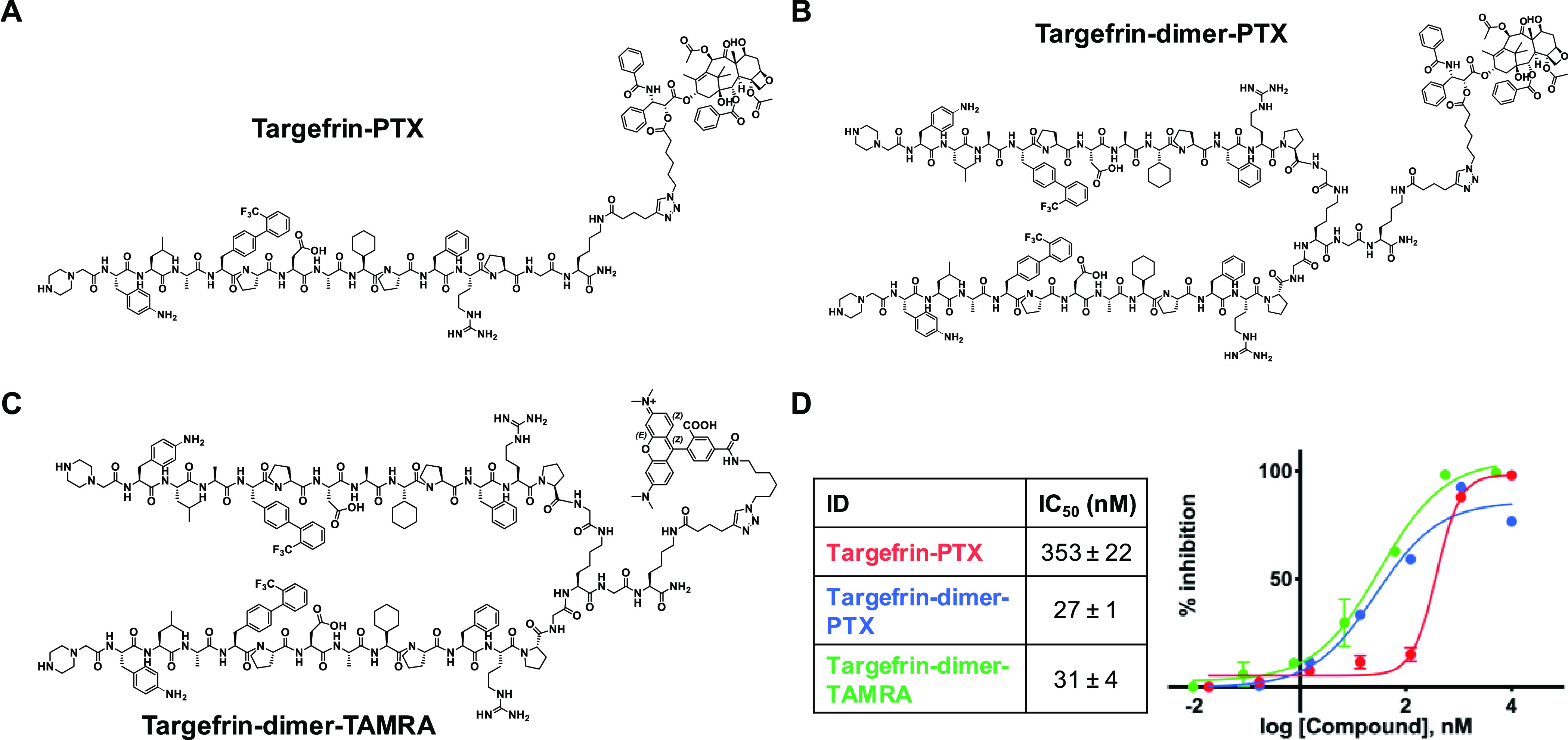
Chemical structure and biochemical activity of targefrin-conjugated agents. Chemical structures of (A) targefrin conjugated to paclitaxel (targefrin-PTX), (B) dimeric version of targefrin conjugated to paclitaxel (targefrin-dimer-PTX), (C) dimeric version of targefrin conjugated to the 5-carboxytetramethylrhodamine-azie dye (targefrin-dimer-TAMRA). (D) DELFIA displacement dose–response curves comparing targefrin-PTX, targefrin-dimer-PTX, and targefrin-dimer-TAMRA, with their respective IC_50_ values.

To assess the utility of targefrin and targefrin-dimer as carriers for targeted delivery, we synthesized and tested drug conjugates including the chemotherapeutic agent paclitaxel and the fluorescent dye TAMRA (Figure 6). The synthesis of these agents followed our previously described “click chemistry” linker that allows for an efficient incorporation in dimeric or monomeric agents of virtually any drugs or imaging reagents (Supporting Information Figures S5, S6, and S7).^1,8^ Conjugation of the dimeric-agents with TAMRA or paclitaxel did not significantly alter their binding properties for isolated EphA2-LBD (Figure 6), while a more significant loss in binding affinity was observed with the targefrin-monomer-PTX, perhaps due to the short linker chosen.

**Figure 7:**
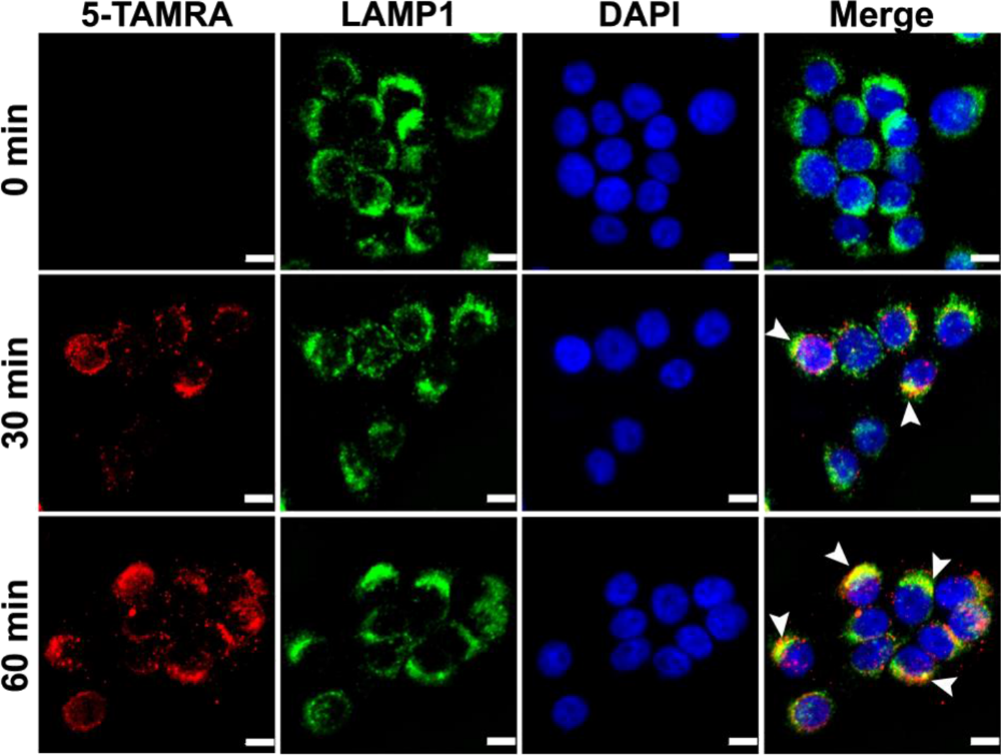
Targefrin-dimer-TAMRA is internalized in EphA2-expressing cells. BxPC3 cells were treated with 100 nM targefrin-dimer-TAMRA for 0, 30, and 60 min. Upon binding of the agent, EphA2 was internalized and targeted to the lysosomes as shown by colocalizations of 5-TAMRA and LAMP1 (arrowheads). Nuclei were labeled in blue. Scale bar = 10 μm.

**Figure 8:**
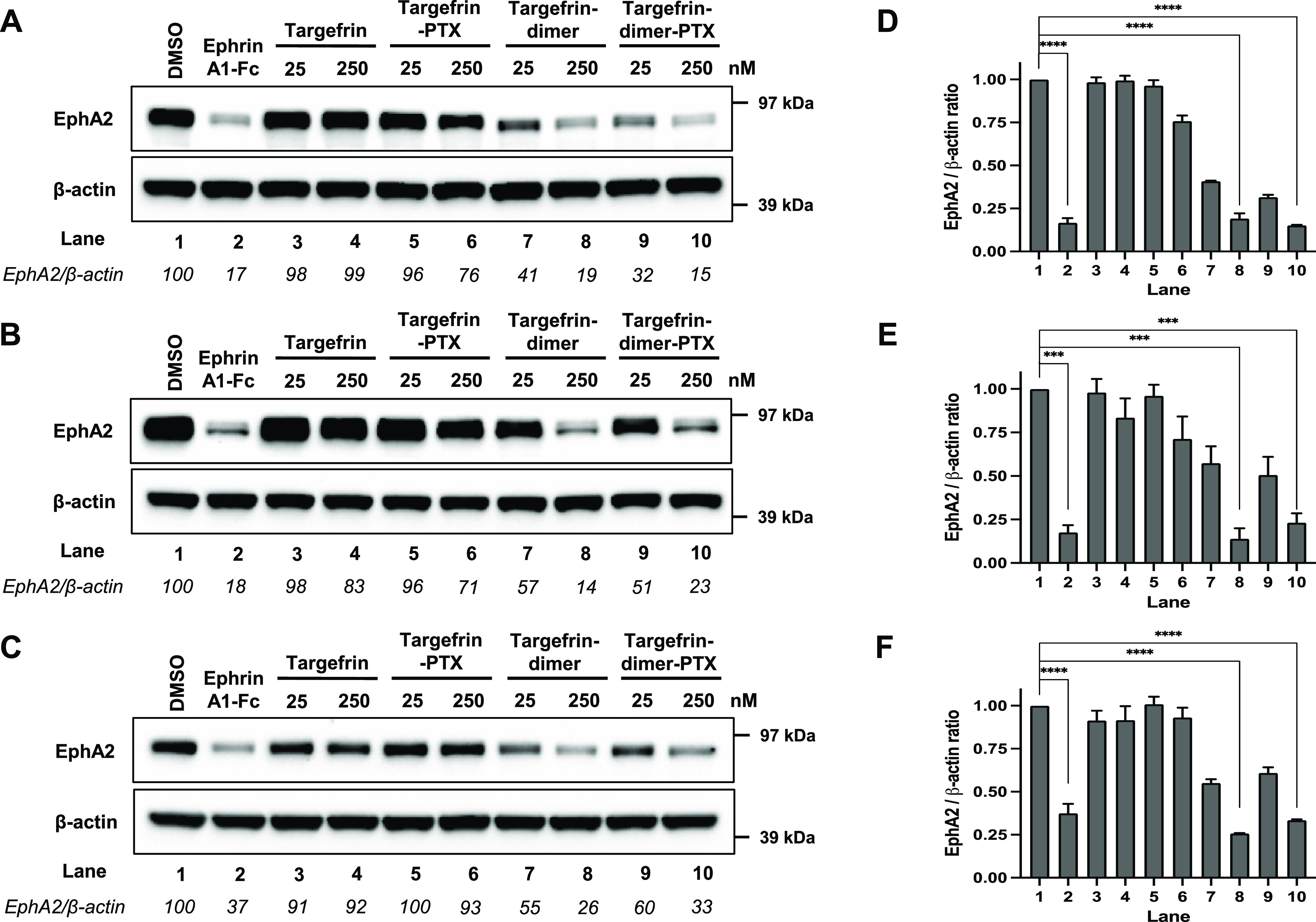
. Targefrin-dimer-PTX retain its ability to cause EphA2 degradation in pancreatic cancer cell lines. (A–C) Western blot images of BxPC3, PANC-1, and MIA PaCa-2 cells, respectively, in which cells were starved for 1 h and treated with 2 μg/mL ephrinA1-Fc or the indicated doses of targefrin, targefrin-PTX, targefrin-dimer, and targefrin-dimer-PTX for 3 h. (D–F) Densitometry analyses of (A–C), respectively. EphA2/β-actin ratios were normalized by designating the EphA2 expression from the DMSO control condition as 100% for (A–C) or 1 for (D–F). ***p < 0.001, ****p < 0.0001, as determined by a one-way analysis of variance using Dunnett’s post-test analysis.

Immunofluorescence microscopy data with BxPC3 cells demonstrated punctuated cytoplasmic fluorescence that co-localized with the lysosomal marker LAMP-1 in the targefrin-dimer-TAMRA treated cells (Figure 7), confirming an EphA2-specific lysosomal internalization event triggered by the agonistic agents. Indeed, targefrin-dimer-PTX retained its ability to cause EphA2 degradation in all three pancreatic cancer cell lines tested (Figure 8).

On the contrary, and as expected, targefrin-monomer-PTX alone did not cause receptor internalization. These data clearly identify targefrin as a potent EphA2-LBD binding agent with antagonistic activity, while targefrin-dimer displayed a similar potent affinity for the isolated EphA2-LBD, but it also displayed potent EphA2 degradation activity in pancreatic cancer cells.

**Figure 9:**
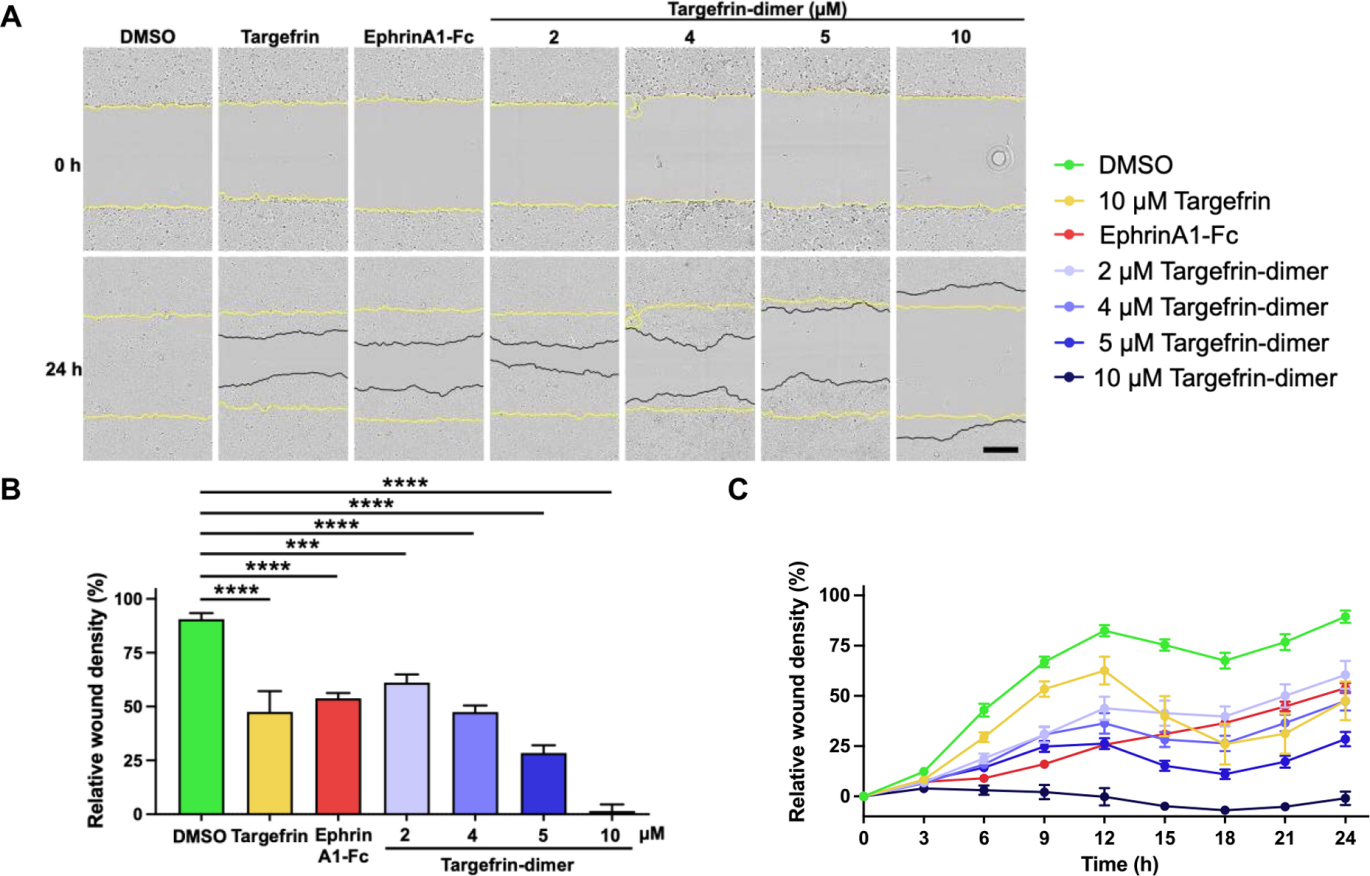
Targefrin-dimer significantly inhibits pancreatic cancer cell migration. (A) Cell migration assay of BxPC3 treated with 2 μg/mL ephrinA1-Fc and 10 μM targefrin or the indicated doses of targefrin-dimer. Plates were imaged every 3 h for 24 h. The yellow lines displayed initial scratches made at 0 h, while the black lines displayed the location that the cells had migrated to after 24 h. (B) Targefrin-dimer significantly inhibited cell migration at 24 h in a dose-dependent manner as shown by decreases in relative wound density. Data relative to the 12 h time point are reported as Supporting Information Figure S10. (C) Time–response curves showed the effects of the agents on wound closure over a period of 24 h. ***p < 0.001, ****p < 0.0001, as determined by a one-way analysis of variance using Dunnett’s post-test analysis. Scale bar = 250 μm.

Finally, to determine whether our EphA2 agonistic agents prevent cell motility of pancreatic cancer cells, we conducted a cell migration assay using the scratch wound method as detected with the time-lapsed live cell analysis (IncuCyte S3, Sartorius) of the pancreatic cancer cell line, BxPC3. We recently reported that in BxPC3, knocking out EphA2 alone resulted in markedly decreased cell migration in this assay.^34^ Similarly, treatment of the BxPC3 cell with increasing concentrations of targefrin-dimer significantly suppressed cell migration (Figure 9). These data conclude that targefrin and targefrin-dimer are potent antagonistic and agonistic EphA2 agents, respectively.

### In Vivo Pharmacology and Mouse Xenograft Studies

Preliminary pharmacokinetic studies with targefrin-dimer were conducted after administration of a single dose of the agent via the tail vein at 50 mg/kg and measuring plasma drug concentration over time (Supporting Information Figure S11). The data show that the agent reaches a C_max_ well above the required 100–200 nM to induce EphA2 degradation in cell and an estimated *t*1/2 ∼ 15 h, suggesting that lower drug concentrations could be used for subsequent in vivo efficacy studies. Blood chemistry analyses after this high dose of targefrin-dimer did not reveal any significantly altered values (Supporting Information Figure S13). In an additional preliminary in vivo toxicity study, Balb/C mice were administered with repeated doses (daily for 5 days) of PTX (8 mg/kg), targefrin-dimer (50 mg/kg), or targefrin-dimer-PTX (50 mg/kg); hence, each group was administered with equivalent doses of PTX. 2 of 3 mice receiving PTX were found dead after the second dose, while the remaining mouse looked lethargic and was found dead by day 5. On the contrary, no adverse signs of toxicity were noted in the targefrin-dimer or the targefrin-dimer-PTX-treated groups (mice in the latter group looked lethargic after day 1 but recovered). Body weight was monitored during the experiment (Supporting Information Figure S14). These preliminary data suggest that targefrin is well tolerated and that it could selectively deliver PTX to EphA2-expressing tumor cells.

**Figure 10:**
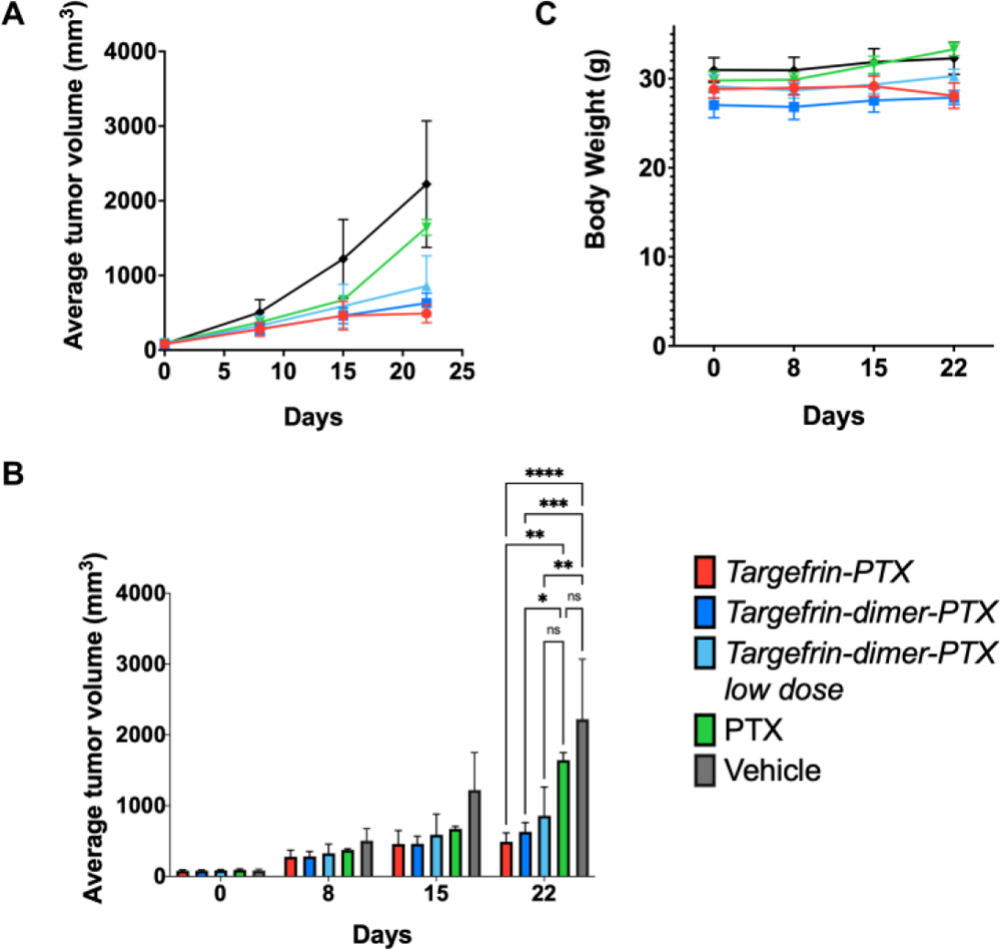
PTX-conjugated suppressed tumor growth in a tumor xenograft with MIA-PaCa-2 cells. (A) Five groups of 5 mice bearing preestablished MIA-PaCa-2 tumors were treated for 22 days with vehicle control alone, paclitaxel (PTX; 2.5 mg/kg), targefrin-PTX (10 mg/kg which is equivalent to 2.5 mg/kg of PTX), targefrin-dimer-PTX (17 mg/kg which is equivalent to 2.5 mg/kg of PTX), and a lower dose of targefrin-dimer-PTX (10 mg/kg which is equivalent to 1.5 mg/kg of PTX). The tumor volume is reported as average ± SE. (B) Average tumor volume for each group of treatment, measured at 0, 8, 15, and 22 days. *p = 0.03, **p < 0.01, *** *p* = 0.0001, **** *p* < 0.0001, as determined by a two-way analysis of variance using Tukey’s post-test analysis. (C) The average body weight ± SE is reported for each of the five groups of treatment at 0, 8, 15, and 22 days. For all the graphs, the vehicle is reported in black, PTX in green, targefrin-dimer-PTX at lower dose in light blue, targefrin-dimer-PTX in blue, and targefrin-PTX in red.

Hence, to further assess the ability of the drug conjugates to direct chemotherapy to pancreatic cancer in vivo, we assessed the ability of the agents to suppress tumor growth in a tumor xenograft with MIA PaCa-2 cells. MIA PaCa-2 cells (1.0 × 10^7^ cells/mouse), in 100 μL of PBS, were first injected into the right flank of five nu/nu mice to obtain tumor stock fragments. Subsequently, a 1 mm^3^ MIA PaCa-2 tumor fragment was grafted in the right flank of each of 25 mice, tumor growth was measured by calipers 18 days after tumor implantation, and mice were grouped to receive treatments on days 1, 4, 8, 11, 15, and 18. The agents were dissolved in a formulation consisting of 80% PBS, 10% Tween 80, 10% ethanol. Five groups received either vehicle control alone, paclitaxel (PTX; 2.5 mg/kg), targefrin-PTX (10 mg/kg which is equivalent to 2.5 mg/kg of PTX), targefrin-dimer-PTX (17 mg/kg which is equivalent to 2.5 mg/kg of PTX), and a lower dose of targefrin-dimer-PTX (10 mg/kg which is equivalent to 1.5 mg/kg of PTX). Both targefrin-PTX and targefrin-dimer-PTX displayed a significant antitumor effect compared to both the untreated group and the PTX-treated group (Figure 10). Moreover, even the group treated with a sub-stoichiometric dose of PTX became more effective than the PTX-treated group (Figure 10), despite the fact that standard deviation on the PTX-treated group is too large to assess significance. The data collectively suggest that the agent is capable of delivering the drug to EphA2-expressing tumors.

## Discussion and Conclusions

In recent years, we have witnessed increasing efforts to target EphA2 by various strategies for the development of novel therapeutics.^43^ These include computational docking strategies,^31,44−46^ NMR-based screening,^40,47,48^ high-throughput screening,^49^ and phage display screening,^39^ and these efforts resulted in potential small-molecule compounds^31,44,8^ or EphA2/ephrin antagonists.^45,46,49−52^ However, in our opinion, none of these cited agents are ripe to be used as potential therapeutics. On the contrary, mAbs have been proposed to target EphA2 but did not perform well in the clinic with reduced selectivity or longer half-life that results in accumulation of the agent in undesired tissues.^53^ Indeed, a very recent phase I clinical study aimed at evaluating the biodistribution of DS-8895a, an anti-EphA2 antibody, in patients with advanced EphA2 positive cancers.^54^ Although encouragingly no treatment-related toxicities were reported, DS-8895a had limited therapeutic efficacy likely due to the observed low tumor uptake, causing halting of any further development of DS-8895a.^54^

**Figure 11:**
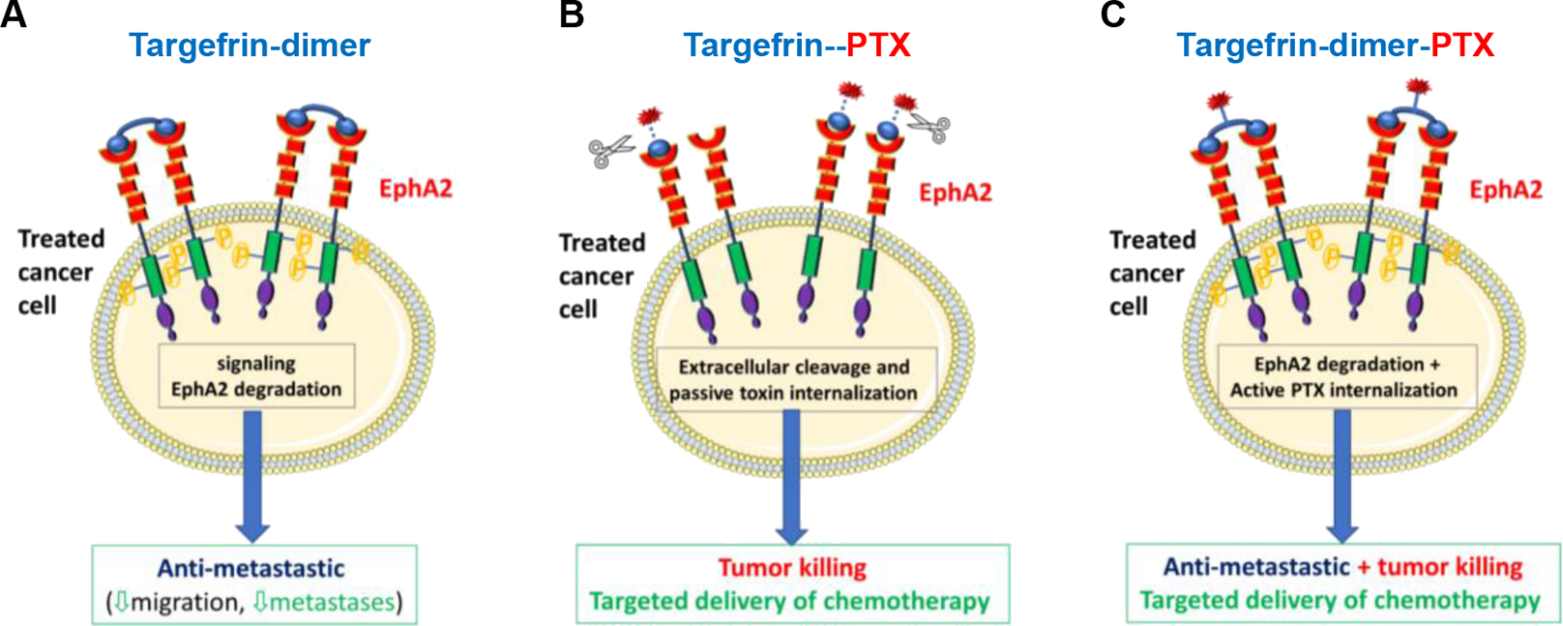
(A) Targefrin-dimer mimics the natural ephrinA1 ligands and cause EphA2 degradation, suppressing metastases. (B) Targefrin-PTX could deliver PTX to cancer cells by virtue of accumulating the toxin on EphA2-expressing metastatic pancreatic cancer cells. This requires specific linker cleavage by extracellular proteins for the toxin to be released and then needs to passively diffuse in tumor cells. (C) When PTX is conjugated to targefrin-dimer, however, EphA2 degradation and pro-metastasis signaling are suppressed. Simultaneously, the toxin is actively transported in EphA2-expressing tumor cells, actively killing primary and metastatic cancer cells.

More recently, Bicycle Therapeutics reported on a peptide antagonist binding to EphA2-LBD that binds with a dissociation constant in the low nanomolar range.^55^ The antagonistic agent was conjugated with monomethyl auristatin linked by a cathepsin cleavable linker, and it is currently in phase I clinical trials (clinicaltrials.gov/ct2/show/NCT04180371). While this agent holds great promise for the first translation of an EphA2-targeting agent into a possible therapeutic, targefrin and targefrin-dimer offer valid alternative strategies to the Bicycle Therapeutics agent. First, targefrin has a similar affinity to the Bicycle Therapeutics compound for EphA2 but possesses reduced molecular weight, presumably enhancing its tissue penetration; second, targefrin-dimer induces active internalization of the receptor functions as an effective EphA2 degrader; hence, it could be deployed as an effective EphA2-based therapeutic to suppress cell migration (Figure 9) as an alternative to agonistic antibodies. Hence, we envision that targefrin-dimer could be deployed as an EphA2 degrader to suppress the metastatic behavior of cancer cells (Figure 11), as we had recently demonstrated with an earlier agent 135H12 in a sworthotopic model of prostate cancer.^33^

Moreover, in drug conjugates, the active internalization induced by targefrin-dimer does not require extracellular linker cleavage and passive diffusion of the cargo, potentially increasing the distribution of the chemotherapeutic agent to EphA2-expressing tumor cells. Indeed, we observed that an earlier dimeric EphA2-targeting agent conjugated with paclitaxel induced a marked reduction of circulating tumor cells in tumor-bearing mice.^8^ Here, we also observed that a sub-therapeutic dose of paclitaxel is effective in reducing tumor volume when conjugated to both monomeric and dimeric versions of targefrin (Figure 10).

Together with our reported preliminary toxicity and pharmacokinetics studies (Supporting Information Figures S9, S13, S14), we suggest that the dimer could be deployed as a single agent or in combination with standard of care to suppress EphA2 in cancer cells. In addition, preliminary studies with drug conjugates should encourage further evaluations with such agents, particularly when conjugated to the targefrin-dimer, exploiting the active internalization provided by the agent to EphA2-overexpressing tumors.

In conclusion, the agents reported herein open the way to a wide range of opportunities for the development of EphA2-targeting therapeutics, ranging from more effective PDCs to the development of diagnostics, or for devising more effective combination therapies targeting tumor metastases.

## Experimental Section

### Chemistry

#### General

All reagents and solvents were obtained from commercial sources, including fmoc-protected amino acids and resins for solid-phase synthesis. All the peptides were synthesized in house by standard microwave-assisted Fmoc peptide synthesis protocols on Rink amide resin using a Liberty Blue peptide synthesizer (CEM). For each coupling reaction, 3 equiv of Fmoc-AA, 3 equiv of DIC, and 1 equiv of OximaPure in 4.5 mL of DMF were used. The coupling reaction was allowed to proceed for 5 min at 90 °C in the microwave reactor. Fmoc deprotection was performed by treating the resin-bound peptide with 20% N-methylpiperidine in DMF (2 × 3 mL) for 3 min at 90 °C. Peptides were cleaved from the resin with a cleavage cocktail containing TFA/TIS/H_2_O/phenol (94:2:2:2) for 5 h (Supporting Information Figure S1). The cleaving solution was filtered from the resin, and the peptides were precipitated in Et_2_O, centrifuged, and dried in a high vacuum. Solution ^1^H NMR was used to check the concentration, and spectra were recorded on a Bruker Avance III 700 MHz. High-resolution mass spectral data were acquired on an Agilent LC-TOF instrument. RP-HPLC purifications were performed on a JASCO preparative system equipped with a PDA detector and a fraction collector controlled by a ChromNAV system (JASCO) on a XTerra C18 10 μ 10 × 250 mm (Waters). The purity of tested compounds was assessed by HPLC using an Atlantis T3 3 μm 4.6 × 150 mm^2^ column (H2O/ACN gradient from 5 to 100% in 45 min). All compounds have a purity >95%.

#### Preparation of Dimeric Agents and Targefrin-Dimer

The preparation of the dimeric agents was done following the procedure described above but doubling the equivalents for each coupling and introducing an Fmoc-Lys(Fmoc)-OH as the first amino acid of the sequence as illustrated in Supporting Information Figure S2.

#### Preparation of Targefrin-Motif and Targefrin-Dimer-Motif

For the preparation of the targefrin-motif and targefrin-dimer-motif, we introduced as first amino acid, coupled to a Rink Amide resin, a Fmoc-Lys(ivDde)-OH amino acid. Subsequently, the peptides were grown following a solid-phase synthetic scheme similar to what we previously described. Upon completion of synthesis, the fully protected peptide on a Rink amide resin was treated with 4% solution of hydrazine in DMF (3 × 5 mL, each 30 min) to remove the ivDde-protecting group and was subsequently washed with DMF (3 × 5 mL). This was followed by a coupling with 3 equiv of 5-hexynoic acid in the presence of 3 equiv of HATU, 3 equiv of OximaPure, and 5 equiv of DIPEA in 1 mL of DMF for 1 h at room temperature. The resin was then washed with DMF (3 × 5 mL) and DCM (3 × 5 mL), dried under vacuum, and cleaved with a cleavage cocktail containing TFA/TIS/H_2_O/phenol (94,2:2:2) for 5 h. Synthetic scheme for targefrin-motif and targefrin-dimer-motif is reported in Supporting Information Figures S3 and S4, respectively.

#### Preparation of Targefrin-PTX, Targefrin-Dimer-PTX, and Targefrin-Dimer-TAMRA

Crude targefrin-motif was dissolved with 1 equiv of PTX-Azide into 4 mL of a 4:1 DMSO:water stirring solution in the presence of 50 μL of CuSO_4_ 1 M and 50 μL of sodium ascorbate 1 M at room temperature for 48 h Supporting Information Figure S5. Targefrin-dimer-PTX was obtained as described above but using a crude targefrin-dimer-motif as the starting point (Supporting Information Figure S6). Targefrin-dimer-TAMRA was obtained as described before but using 1 equiv of 5-TAMRA-azide instead of PTX-azide (Supporting Information Figure S6). Analytical HPLC traces for key compounds are reported as Supporting Information Figure S8, and mass spectrometry data for all peptide synthesis are reported in Supporting Information Table S1.

### ITC Measurements

To obtain information about the dissociation constant (*K*_d_) and thermodynamics of binding of our EphA2-targeting agents, we tested the compounds by ITC performed using the Affinity ITC Autosampler from TA Instruments (New Castle, DE) against EphA2-LBD. The titrations were performed in a reverse fashion by titrating the protein into the ligand solution. All titrations were performed dissolving both the agents and the targeting protein in 25 mM Tris at pH 7.5, 150 mM NaCl, at 25 °C with a final DMSO concentration of 1%. The syringe was filled with a 200 μM solution of EphA2-LBD, EphA3-LBD Chimera, or EphA4-LBD, performing 20 injections of 2.5 μL each into the cell containing a 10 μM solution of the compounds. The injections were made at 200 s intervals with a stirring speed of 75 rpm. The solutions were kept in the autosampler at 4 °C. The analysis of the data was performed by the NanoAnalyze software (TA Instruments, New Castle, DE) and subsequently exported into Microsoft Excel.

### DELFIA Displacement Assays

To test the activity of the dimeric and monomeric agents, a solution of 100 μL of 1 μM of 123B9-Biotin^1^ or 100 nM of agent PiperazineAcAcid-YSA-(2MeBip)-PDS-Chg-PFRP-GK(Biotin LC) was added to each well of 96-well streptavidin-coated plates, respectively, and incubated for 2 h. Plates were then washed three times. Subsequently, a mixture containing 11 μL of EphA2 protein and a serial dilution of the test compounds was added to each well and incubated with a solution containing 89 μL of Eu-N1-labeled anti-6x-His antibody (PerkinElmer) for 1 h. At the end of the incubation period, plates were washed three times and incubated with DELFIA enhancement solution (PerkinElmer) for 10 min. The final concentrations of the EphA2 protein used to test the activity of dimeric and monomeric agents were 71.2 and 10 nM, respectively. The antibody concentrations in a solution of 89 μL used to test the dimeric and monomeric agents were 4.17 and 3.13 nM, respectively. EphA2 protein, biotinylated peptides, and antibody were prepared in DELFIA assay buffer (PerkinElmer). Fluorescence measurements were taken with the VICTOR X5 microplate reader (ex/em of 340/615 nm), normalized to DMSO wells, and reported as percent inhibition. Prism 9 (GraphPad) was used to calculate IC_50_ values.

### Cell Lines, Cell Culture, and Antibodies

BxPC3, MIA PaCa-2, and PANC-1 cell lines were purchased from the American Type Culture Collection (ATCC). BxPC3 and PANC-1 cells were cultured in RPMI-1640 medium and DMEM, respectively, and supplemented with 10% fetal bovine serum (FBS). MIA PaCa-2 cells were cultured in DMEM supplemented with 10% FBS and 2.5% horse serum. Cells were maintained at 37 °C in a humidified incubator with 5% CO_2_. Anti-EphA2 antibody (#374400), HRP-conjugated goat anti-mouse secondary antibody (#31432), and Alexa Fluor 488-conjugated goat anti-rabbit secondary antibody (#A-11034) were purchased from ThermoFisher Scientific. Anti-β-actin antibody (#sc-69879) was purchased from Santa Cruz Biotechnology, and anti-LAMP1 antibody (#9091) was purchased from Cell Signaling Technology.

### Immunofluorescence

BxPC3 cells were plated on the coverslips overnight. Cells were serum starved for 1 h and treated with 100 nM targefrin-dimer-TAMRA for 0, 30, and 60 min. Cells were then fixed with 4% paraformaldehyde for 20 min, permeabilized with 0.2% Triton X-100 for 5 min, blocked with 10% goat serum for 1 h, and incubated with an anti-LAMP1 antibody overnight at 4 °C, followed by incubation with an anti-rabbit secondary antibody conjugated with Alexa Fluor 488 for 1 h at room temperature. VECTASHIELD antifade mounting medium containing DAPI (Vector Laboratories) was added to the coverslips to stain for the nuclei. Images were then acquired using a Zeiss Axiovert 200 M fluorescence deconvolution microscope and processed with a SlideBook software version 6 (Intelligent Imaging Innovations).

### Immunoblotting

After treatments, cells were lysed on ice with a lysis buffer (20 mM Tris, pH 7.4, 120 mM NaCl, 1% Triton X-100, 0.5% sodium deoxycholate, 0.1% SDS, 1% IGEPAL, and 5 mM EDTA, supplemented with a protease inhibitor cocktail and PhosSTOP (Sigma-Aldrich)). Lysates were then centrifuged at 16,000 × *g* for 20 min at 4 °C, and supernatants were collected. Protein determination was done using the Pierce BCA Protein Assay Kit (ThermoFisher Scientific) according to the manufacturer’s protocol. Samples were prepared and loaded onto 4–12% NuPAGE Bis-Tris precast gels prior to being transferred onto PVDF membranes. Blots were blocked with 5% nonfat milk for 1 h at room temperature and incubated with monoclonal EphA2 or actin antibodies overnight at 4 °C followed by an incubation with an anti-mouse HRP-conjugated antibody for 1 h at room temperature. The Clarity Western ECL kit (BIO-RAD) was added to the blots, and images were captured with the ChemiDoc imaging system (BIO-RAD) and analyzed using ImageJ software. Uncropped images of western blots, including repeated experiments, are available in Supporting Information Figure S10.

### Cell Migration Assays

BxPC3 cells were seeded in the IncuCyte ImageLock 96-well plates (Sartorius) so that they were approximately at 95–100% confluency by the time of the treatment. Wounds were then made on a monolayer of cells using the WoundMaker (Sartorius) followed by two washes with PBS. Cells were subsequently treated with 2 μg/mL ephrinA1-Fc (R&D Systems) or the test agents, and the plates were imaged every 3 h with the IncuCyte S3 live-cell analysis system (Sartorius). The percentage relative wound density was quantified using the IncuCyte cell migration software module.

### In Vivo Pharmacokinetics, Toxicity, and Xenograft Studies

The in vivo efficacy experiment was conducted at AntiCancer, Inc. (San Diego). For the xenograft study, 35 male nu/nu mice (AntiCancer Inc., San Diego), 8–10 weeks of age, were used, consisting of 25 mice for randomization and 10 extra mice. All mice were kept in a barrier facility on a high efficacy particulate air (HEPA)-filtered rack under standard conditions of 12 h light/dark cycles. Animal studies were performed with an AntiCancer Institutional Animal Care and Use Committee (IACUC)-protocol specially approved for this study and in accordance with the principles and procedures outlined in the National Institutes of Health Guide for the Care and Use of Animals under Assurance Number A3873-1. Autoclaved, acidified water (pH 2.5–3) was supplied ad libitum to all animals. Cryogenic vials containing MIA PaCa-2 pancreatic cancer cells were thawed from liquid nitrogen storage and expanded for in vitro cell culture to prepare subcutaneous stock tumor for subsequent flank tumor-fragment implantation. MIA PaCa-2 cells were maintained in DMEM supplemented with 10% heat-inactivated fetal bovine serum and 1% penicillin and cultured at 37 °C in a 5% CO2 incubator. Hence, MIA PaCa-2 cells (1.0 × 107 cells/mouse), in 100 μL of PBS, were injected into the right flank of five male nu/nu mice. After mice were put under anesthesia using a ketamine solution, an approximate 5 mm incision was made on the back of nude mice. After making a space under the skin of the right flank, a 1 mm^3^ MIA PaCa-2 tumor fragment, prepared from stock, was inserted. The incision was closed with a 5–0 PDS-II suture. 18 days after tumor implantation (Day 0), tumors were measured by calipers using the formula: (Tumor volume) = (Length) × (Width) × (Width) × 1/2. 25 out of 35 mice were randomized into five treatment groups of 5 mice, with no significant difference in tumor volume between the groups. All treatment agents (dissolved in 100 μL of formulation composed of 80% PBS, 10% Tween 80, 10% ethanol) were administered by tail vein injection twice per week for 3 weeks for a total of 6 injections. Treatment was begun the day after randomization (day 1), and mice received agents or vehicle control on days 1, 4, 8, 11, 15, and 18. Tumor volume and body weight were measured weekly. The study was terminated 22 days after the initiation of the treatment.

### Molecular Modeling

Molecular models were analyzed using MOE 2022.02 (Chemical Computing Group). The model of targefrin in complex with EphA2-LBD was obtained by modifying and properly minimizing the crystal structure of our previous agent with EphA2-LBD (PDB-ID 6B9L).

## Supporting Information Available

Molecular formula strings for each final compound with their enzyme and cellular IC_50_ as well as mouse microsomal stability (CSV)
Mass spectroscopy data of investigated compounds; tested agents with 4-phenyl-*L*-phenylalanine fixed in position 4 and relative *K*_d_ values (nM) from ITC; tested agents with 4-(2-methylphenyl)-*L*-phenylalanine fixed in position 4 and relative *K*_d_ values (nM) from ITC; synthetic scheme used to prepare monomeric peptides, dimeric peptides, targefrin-motif, targefrin-dimer-motif, targefrin-PTX, targefrin-dimer-PTX, targefrin-dimer-TAMRA; HPLC traces of keys compounds; pharmacokinetics studies; uncropped western blots that generated the data in Figures 4, 5, and 8; cell migration assay of BxPC3 at 12 h (relative to Figure 9); cell viability assay of MIA PaCa-2 treated with targefrin or ephrinA1-Fc; chemistry analysis for mice treated with targefrin-dimer; and repeated dose toxicity studies with targefrin-dimer-PTX versus PTX alone (PDF)
Coordinates for the homology model of targefrin in complex with EphA2-LBD based on the X-ray structure of the complex with one of our earlier peptides (PDB ID 6B9L) (PDB)

## Supplementary Material

jm2c01391_si_002.pdf

jm2c01391_si_003.pdb

## Abbreviations

EphA2-LBD: ephrin type-A receptor 2 ligand-binding domain
ITC: isothermal titration calorimetry
