# Supplementary material for: Targefrin: A Potent Agent Targeting the Ligand Binding Domain of EphA2
Source: J Med Chem. 2022 Nov 4;65(22):15443–56. doi: 10.1021/acs.jmedchem.2c01391 (PMC9706575; doi:10.1021/acs.jmedchem.2c01391)
Supplement: Supplementary file 2 — jm2c01391_si_002.pdf [file jm2c01391_si_002.pdf]

Supplementary Information

Targefrin: a potent agent targeting the ligand binding domain of EphA2

Carlo Baggio[1,2], Parima Udompholkul[1,2], Luca Gambini[1], and Maurizio Pellecchia[1,*]

[1]*Division of Biomedical Sciences, School of Medicine, University of California Riverside, 900 University Avenue, Riverside, CA 92521, USA.*

[2] *These authors contributed equally to this work.*

*Corresponding author: Maurizio Pellecchia, Ph.D. email: Maurizio.Pellecchia@ucr.edu

Table of Contents.

- Supplementary **Table S1** reports mass spectroscopy data of investigated compounds. Page S3
- Supplementary **Table S2** reports tested agents with 4-phenyl-L-phenylalanine fixed in position 4 and relative K_d values (nM) from ITC. Page S5
- Supplementary **Table S3** reports tested agents with 4-(2-Methylphenyl)-L-phenylalanine fixed in position 4 and relative K_d values (nM) from ITC. Page S6
- Supplementary **Figure S1** reports the synthetic scheme used to prepare monomeric peptides. Page S7
- Supplementary **Figure S2** reports the synthetic scheme used to prepare dimeric peptides. Page S8
- Supplementary **Figure S3** reports the synthetic scheme used to prepare targefrin-motif. Page S9
- Supplementary **Figure S4** reports the synthetic scheme used to prepare targefrin-dimer-motif. Page S10
- Supplementary **Figure S5** reports the synthetic scheme used to prepare targefrin-PTX.Page S11
- Supplementary **Figure S6** reports the synthetic scheme used to prepare targefrin-dimer-PTX. Page S12
- Supplementary **Figure S7** reports the synthetic scheme used to prepare targefrin-dimer-TAMRA. Page S13
- Supplementary **Figure S8** reports HPLC traces of keys compounds. Page S14
- Supplementary **Figure S9** reports pharmacokinetics studies. Page S16
- Supplementary **Figure S10** reports uncropped western blots that generated the data in **Figures 4, 5** and **8**. Page S17
- Supplementary **Figure S11** reports cell migration assay of BxPC3 at 12 h (relative to **Figure 9**). Page S20

- Supplementary **Figure S12** reports cell viability assay of MIA PaCa-2 treated with targefrin or ephrinA1-Fc. Page S21
- Supplementary **Figure S13** reports chemistry analysis for mice treated with targefrin-dimer. Page S22
- Supplementary **Figure S14** reports repeated doses toxicity studies with targefrin-dimer-PTX versus PTX alone. Page S23

Table S1. Mass-spectrometry data of investigated compounds. All the compounds were analyzed using an Agilent 6545 QTOF LC/MS instrument.

ID	Sequence	Calcd [M]	Obs. $[M+H]^+$ (m/z)
**147B4 2**	H_2N-YSA-Bip-PDSVPFRP-$CONH_2$	1456.7080	1457.7160
**152B6 3**	H_2N-YSA-(Thyronine)-PDSVPFRP-$CONH_2$	1488.7038	1489.7111
**152A5 4**	H_2N-YSA-(2-MeO-Bip)-PDSVPFRP-$CONH_2$	1486.7245	1487.7335
**152A11 5**	H_2N-YSA-(4-Cl-Bip)-PDSVPFRP-$CONH_2$	1490.6750	1491.6783
**152A12 6**	H_2N-YSA-(2,6DiMeO-Bip)-PDSVPFRP-$CONH_2$	1516.7351	1517.7389
**152B1 7**	H_2N-YSA-(4-Me-Bip)-PDSVPFRP-$CONH_2$	1470.7296	1471.7347
**152B2 8**	H_2N-YSA-(2-Me-Bip)-PDSVPFRP-$CONH_2$	1470.7296	1471.7366
**152C11 9**	H_2N-YSA-(2-CF_3-Bip)-PDSVPFRP-$CONH_2$	1524.7013	1525.7113
**152D1 10**	H_2N-YSA-(2Me-4MeO-Bip)-PDSVPFRP-$CONH_2$	1500.7402	1501.7491
**152A1 11**	MorphAcAcid-YSA-Bip-PDSVPFRP-$CONH_2$	1583.7773	1584.7852
**152A9 12**	PiperazineAcAcid-YSA-Bip-PDSVPFRP-$CONH_2$	1582.7932	1583.7960
**152A7 13**	H_2N-WSA-Bip-PDSVPFRP-$CONH_2$	1479.7299	1480.7402
**152A4 14**	H_2N-(5-OH-Trp)-SA-Bip-PDSVPFRP-$CONH_2$	1495.7248	1496.7326
**152C7 15**	H_2N-1Nal-SA-Bip-PDSVPFRP-$CONH_2$	1490.7347	1491.7431
**152C8 16**	H_2N-2Nal-SA-Bip-PDSVPFRP-$CONH_2$	1490.7347	1491.7432
**152C9 17**	H_2N-(Me-Tyr)-SA-Bip-PDSVPFRP-$CONH_2$	1470.7296	1471.7389
**152C10 18**	H_2N-(2-NO_2-Phe)SA-Bip-PDSVPFRP-$CONH_2$	1485.7041	1486.7136
**152D5 19**	H_2N-(2-NH_2-Phe)-SA-Bip-PDSVPFRP-$CONH_2$	1455.7299	1456.7380
**152D12 20**	H_2N-(pyridyl-Ala)-SA-Bip-PDSVPFRP-$CONH_2$	1441.7143	1442.7207
**152C5 21**	H_2N-Y-L-A-Bip-PDSVPFRP-$CONH_2$	1482.7660	1483.7757
**152D8 22**	H_2N-Y-T-A-Bip-PDSVPFRP-$CONH_2$	1470.7296	1471.7438
**152A8 23**	H_2N-YSA-Bip-PDS-Chg-PFRP-$CONH_2$	1496.7452	1497.7493
**152D6 24**	PiperazAcAcid-(2Nal)-L-A-(2-CF_3-Bip)-PDS-Chg-P-(4Cl-Phe)-RP-$CONH_2$	1784.8457	1785.8530
**152D11 25**	PiperazAcAcid-(2NH_2-Phe)-SA-(2-CF_3-Bip)-PDS-Chg-PFRP-$CONH_2$	1689.8279	1690.8374
**152E1 26**	PiperazAcAcid-(2NH_2-Phe)-L-A-(2-CF_3-Bip)-PDS-Chg-PFRP-$CONH_2$	1715.8800	(z = 3) 573.3014 (z = 2) 858.9478
**Targefrin (152E2) 27**	PiperazAcAcid-(2NH_2-Phe)-L-A-(2-CF_3-Bip)-PD-A-Chg-PFRP-$CONH_2$	1699.8850	(z = 3) 567.9696 (z = 2) 851.4519
**Targefrin-dimer 152E3 28**	[PiperazAcAcid-(2-NH_2-Phe)-L-A-(2-CF_3-Bip)-PD-A-Chg-PFRPG]$_2$-K-$CONH_2$	3625.8848	(z = 5) 726.3855 (z = 4) 907.7288 (z = 3) 1209.9690
**152E5 29**	[PiperazAcAcid-(2-NH_2-Phe)-L-A-(2-CF_3-Bip)-PD-A-Chg-PFRP-(β-Ala)]$_2$-K-$CONH_2$	3653.9161	(z = 5) 731.9890 (z = 4) 914.7353 (z = 3)

			1219.3109
**152E6 30**	[PiperazAcAcid-(2-NH_2-Phe)-L-A-(2-CF_3-Bip)-PD-A-Chg-PFRP-(GABA)]$_2$-K-$CONH_2$	3681.9474	(z = 5) 737.5954 (z = 4) 921.7432 (z = 3) 1228.6543
**Targefrin-PTX 152F1**	PiperazAcAcid-(2-NH_2-Phe)-L-A-(2-CF_3-Bip)-PD-A-Chg-PFRPG-K-(Linker)-PTX	2972.4522	(z = 4) 744.1197 (z = 3) 991.8244 (z = 2) 1487.2337
**Targefrin-dimer-PTX 152E4**	[PiperazAcAcid-(2-NH_2-Phe)-L-A-(2-CF_3-Bip)-PD-A-Chg-PFRPG]$_2$-KGK-(Linker)-PTX	4898.4519	(z = 6) 817.5799 (z = 5) 980.8969 (z = 4) 1225.6179 (z = 3) 1633.8200
**Targefrin-dimer-TAMRA 152E7**	[PiperazAcAcid-(2-NH_2-Phe)-L-A-(2-CF_3-Bip)-PD-A-Chg-PFRPG]$_2$-KGK-(Linker)-TAMRA	4461.3179	(z = 7) 638.3418 (z = 6) 744.3911 (z = 5) 893.0693 (z = 4) 1116.0822
**152B8 31**	PiperazineAcAcid-YSA-Bip-PDS-Chg-PFRP-$CONH_2$	1622.8245	1623.8279
**152D7 32**	PiperazAcAcid-YSA-(Bip)-PDS-Chg-P-(4Cl-Phe)-RP-$CONH_2$	1656.7856	1657.7900
**152D9 33**	PiperazAcAcid-Y-L-A-(Bip)-PDS-Chg-PFRP-$CONH_2$	1648.8766	1649.8886
**152D10 34**	PiperazAcAcid-(2Nal)-SA-(Bip)-PDS-Chg-PFRP-$CONH_2$	1656.8453	1657.8520
**152B12 35**	PiperazineAcAcid-YSA-(2Me-Bip)-PDS-Chg-PFRP-$CONH_2$	1636.8402	1637.8480
**152C12 36**	PiperazAcAcid-(2Nal)-L-A-(2Me-Bip)-PDS-Chg-P-(4Cl-Phe)-RP-$CONH_2$	1730.8740	(z = 2) 866.9478
**152D2 37**	PiperazAcAcid-Y-L-A-(2Me-Bip)-PDS-Chg-P-(4Cl-Phe)-RP-$CONH_2$	1696.8533	1697.8605

Table S2. Tested agents with 4-phenyl-L-phenylalanine fixed in position 4 and relative K_d values (nM) from ITC.

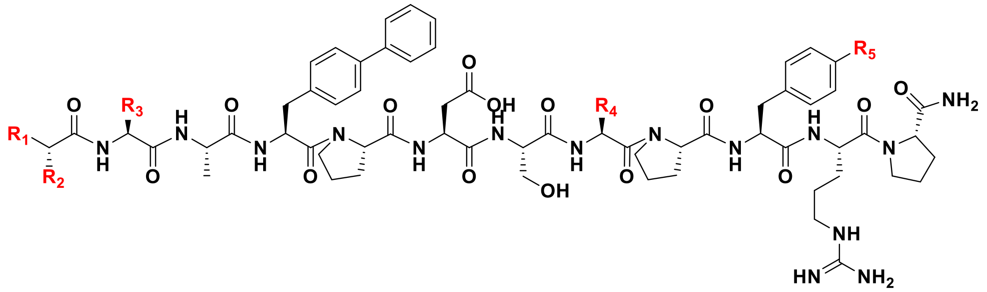

ID	R_1	R_2	R_3	R_4	R_5	Kd (nM) - ITC
**152B8 (31)**	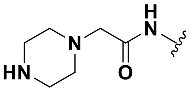	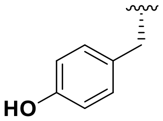	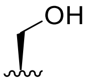	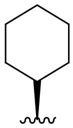	H	54
**152D7 (32)**	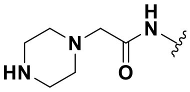	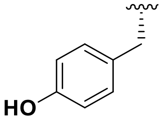	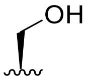	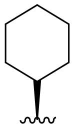	Cl	68
**152D9 (33)**	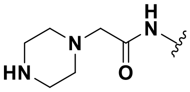	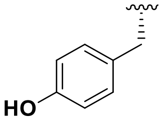	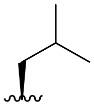	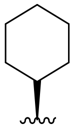	H	39
**152D10 (34)**	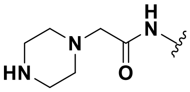	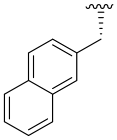	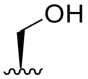	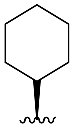	H	74

Table S3. Tested agents with 4-(2-Methylphenyl)-L-phenylalanine fixed in position 4 and relative K_d values (nM) from ITC.

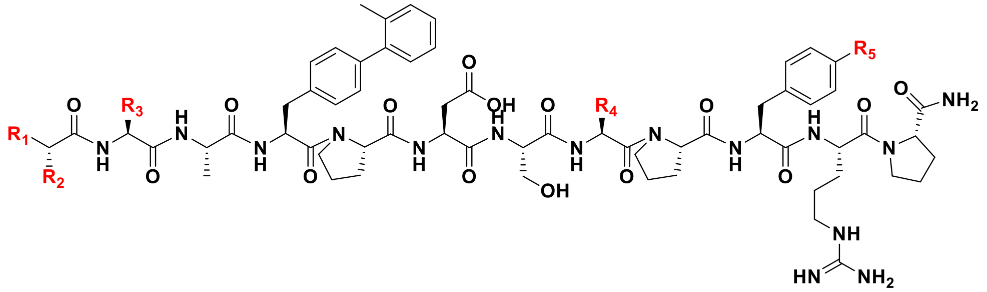

ID	R_1	R_2	R_3	R_4	R_5	Kd (nM) - ITC	IC50 (nM)
**152B12 (35)**	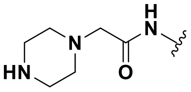	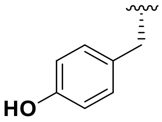	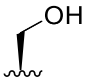	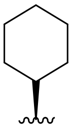	H	8.6	22.7
**152C12 (36)**	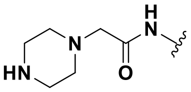	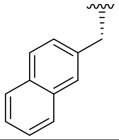	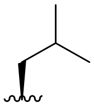	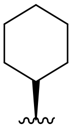	Cl	---	30.8
**152D2 (37)**	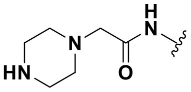	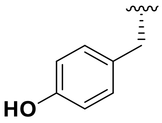	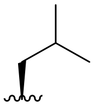	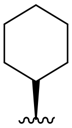	Cl	49	16.5

Figure S1. **Synthetic scheme for the synthesis of *Targefrin***. Conditions: (a) Rink Amide resin + 3 equiv. of Fmoc-Pro-OH, 3 equiv. of DIC, 1 equiv. of OximaPure, in 4.5 mL of DMF. Reaction for 5 min at 90°C in the microwaved-assisted Liberty Blue peptide synthesizer. (b) Fmoc deprotection with 20% N-methylpiperidine in DMF twice for 3 min at 90°C in the microwaved-assisted Liberty Blue peptide synthesizer; (c) Peptides growth using previous conditions on Liberty Blue system; (d) TFA/TIS/water/phenol (94:2:2:2), 5 h, rt.

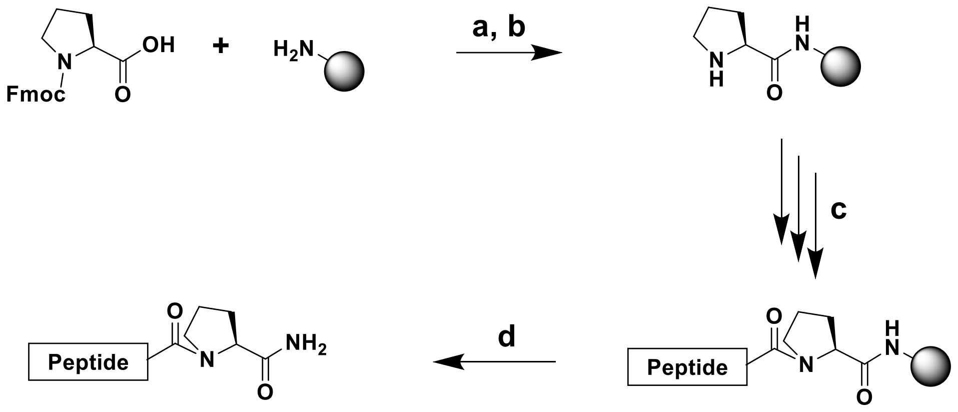

Figure S2. **Synthetic scheme for the synthesis of Targefrin-dimer**. Conditions: (a) Rink Amide resin + 3 equiv. of Fmoc-Lys(Fmoc)-OH, 3 equiv. of DIC, 1 equiv. of OximaPure, in 4.5 mL of DMF. Reaction for 5 min at 90°C in the microwaved-assisted Liberty Blue peptide synthesizer; (b) Fmoc deprotection with 20% N-methylpiperidine in DMF twice for 3 min at 90°C in the microwaved-assisted Liberty Blue peptide synthesizer; (c) Peptides growth using previous conditions but using double equivalents for dimer growth: 6 equiv. of Fmoc-Amino Acid, 6 equiv. of DIC, 2 equiv. of OximaPure, in 4.5 mL of DMF. Reaction for 5 min at 90°C in the microwaved-assisted Liberty Blue peptide synthesizer; (d) TFA/TIS/water/phenol (94:2:2:2), 5 h, rt.

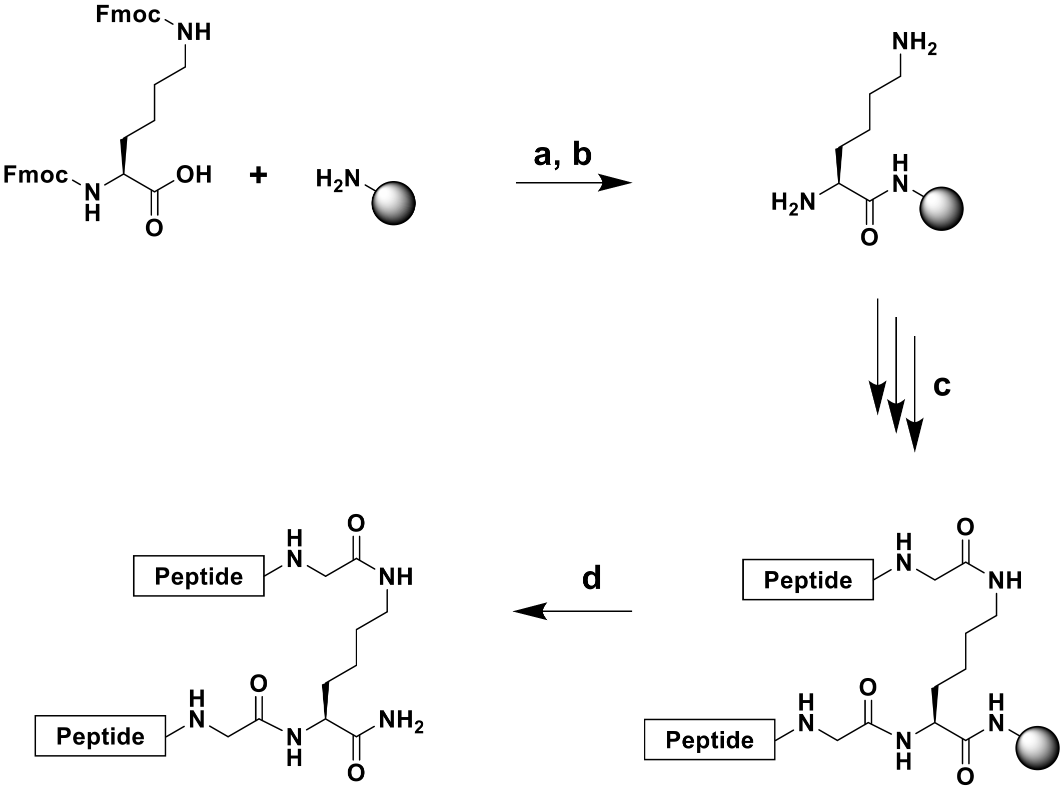

Figure S3. **Synthetic scheme for the synthesis of compound *Targefrin-motif***. Conditions: (a) Rink Amide resin + 3 equiv. of Fmoc-Lys(ivDde)-OH, 3 equiv. of HATU, 3 equiv. of OximaPure, and 5 equiv. of DIPEA in 1 mL of DMF, 1 h, rt; (b) Fmoc deprotection with 20% piperidine in DMF twice; (c) 3 equiv. of Fmoc-Gly-OH, 3 equiv. of HATU, 3 equiv. of OximaPure, and 5 equiv. of DIPEA in 1 mL of DMF, 1 h, rt; (d) Peptides growth using previous conditions or LibertyBlue; (e) ivDde deprotection using 4% N_2H_2 in DMF (3 × 5 mL), rt; (f) 3 equiv. of 5-Hexynoic acid, 3 equiv. of HATU, 3 equiv. of OximaPure, and 5 equiv of DIPEA in 1 mL of DMF, 1 h, at rt; (g) TFA/TIS/water/phenol (94:2:2:2), 5 h, rt.

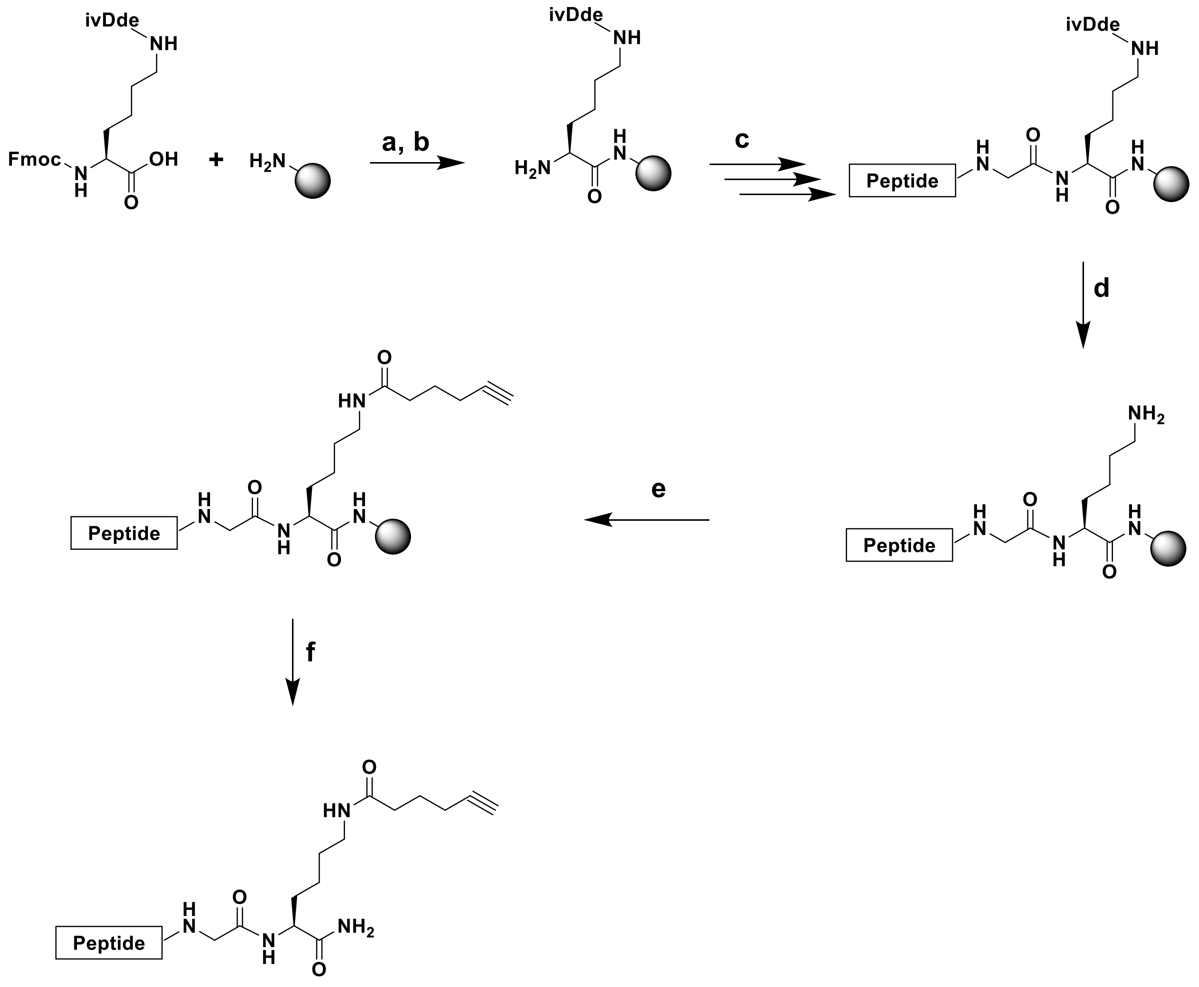

Figure S4. **Synthetic scheme for the synthesis of compound *Targefrin-dimer-motif***. Conditions: **(a)** Rink Amide resin + 3 equiv. of Fmoc-Lys(ivDde)-OH, 3 equiv. of HATU, 3 equiv. of OximaPure, and 5 equiv. of DIPEA in 1 mL of DMF, 1 h, rt. **(b)** Fmoc deprotection with 20% N-methylpiperidine in DMF twice. **(c)** 3 equiv. of Fmoc-Gly-OH, 3 equiv. of HATU, 3 equiv. of OximaPure, and 5 equiv. of DIPEA in 1 mL of DMF, 1 h, rt. **(d)** 3 equiv. of Fmoc-Lys(Fmoc)-OH, 3 equiv. of HATU, 3 equiv. of OximaPure, and 5 equiv. of DIPEA in 1 mL of DMF, 1 h, rt. **(e)** Peptides growth double equivalents for dimer growth: 6 equiv. of Fmoc-Amino Acid, 6 equiv. of DIC, 2 equiv. of OximaPure, in 4.5 mL of DMF. Reaction for 5 min at 90°C in the microwaved-assisted Liberty Blue peptide synthesizer. **(f)** ivDde deprotection using 4% N_2H_2 in DMF (3 × 5 mL), rt; **(g)** 3 equiv. of 5-Hexynoic acid, 3 equiv. of HATU, 3 equiv. of OximaPure, and 5 equiv of DIPEA in 1 mL of DMF, 1 h, at rt; **(h)** TFA/TIS/water/phenol (94:2:2:2), 5 h, rt.

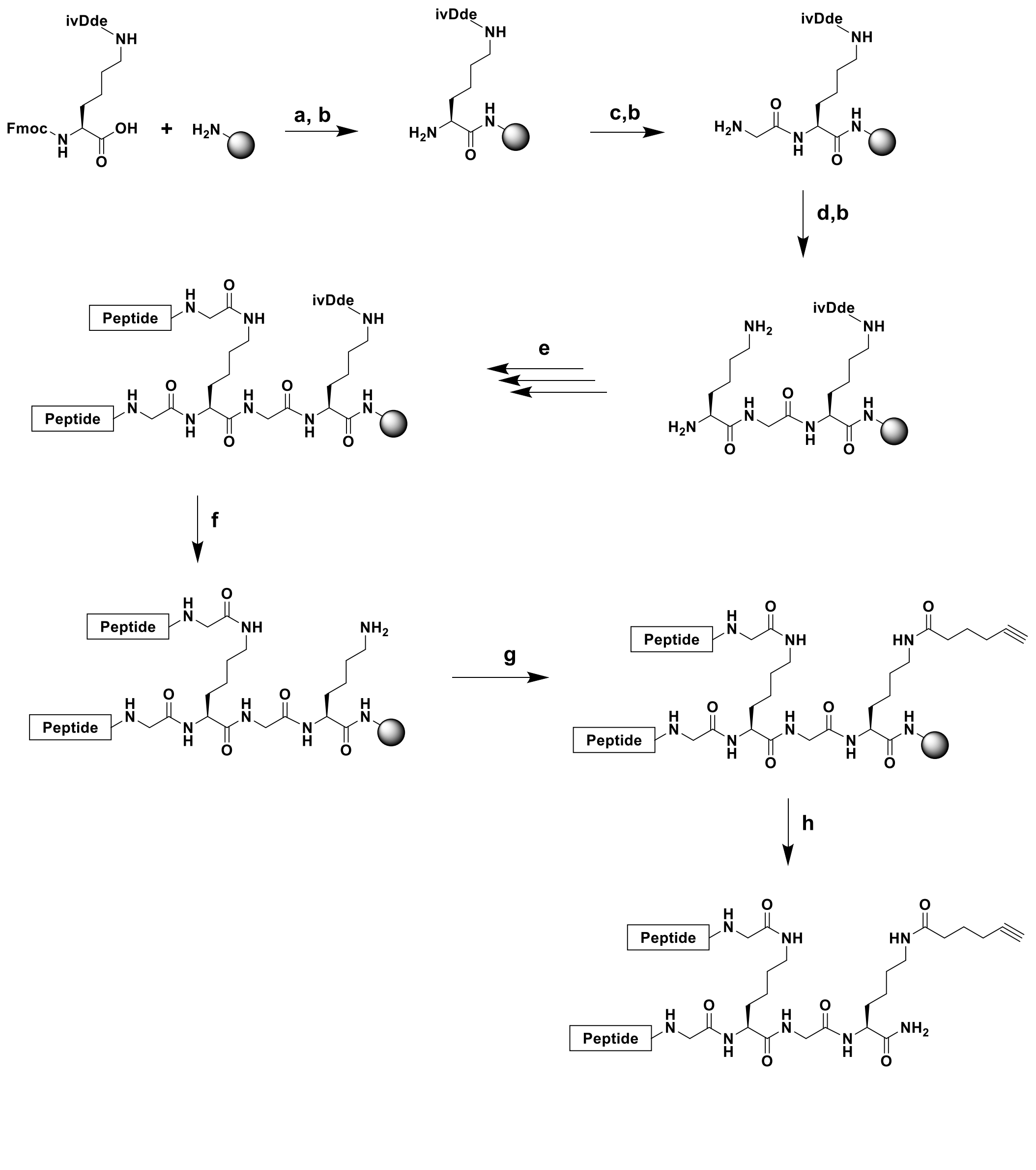

Figure S5. **Synthetic scheme for the synthesis of Targefrin-PTX**. . Conditions: (a) *Targefrin-motif* crude, 1 equiv. of PTX-Azide in 4 mL of 4:1 DMSO:water solution. Add 50 uL of $CuSO4$ 1M and 50 uL of Sodium Ascorbate 1M. Mix at rt for 48 h.

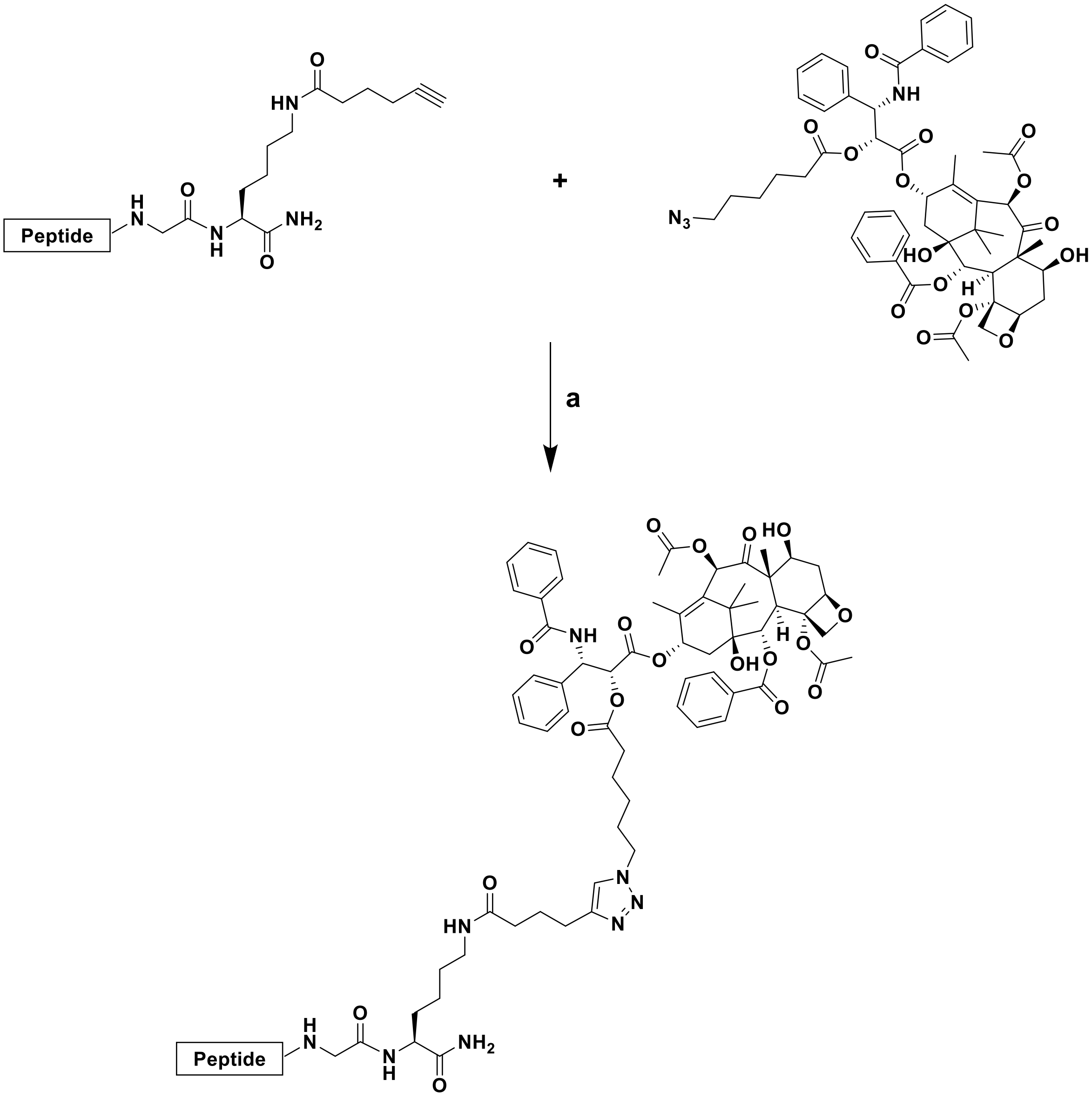

Figure S6. **Synthetic scheme for the synthesis of Targefrin-dimer-PTX**. . Conditions: **(a)** *Targefrin-dimer-motif* crude, 1 equiv. of PTX-Azide in 4 mL of 4:1 DMSO:water solution. Add 50 uL of $CuSO_4$ 1M and 50 uL of Sodium Ascorbate 1M. Mix at rt for 48 h.

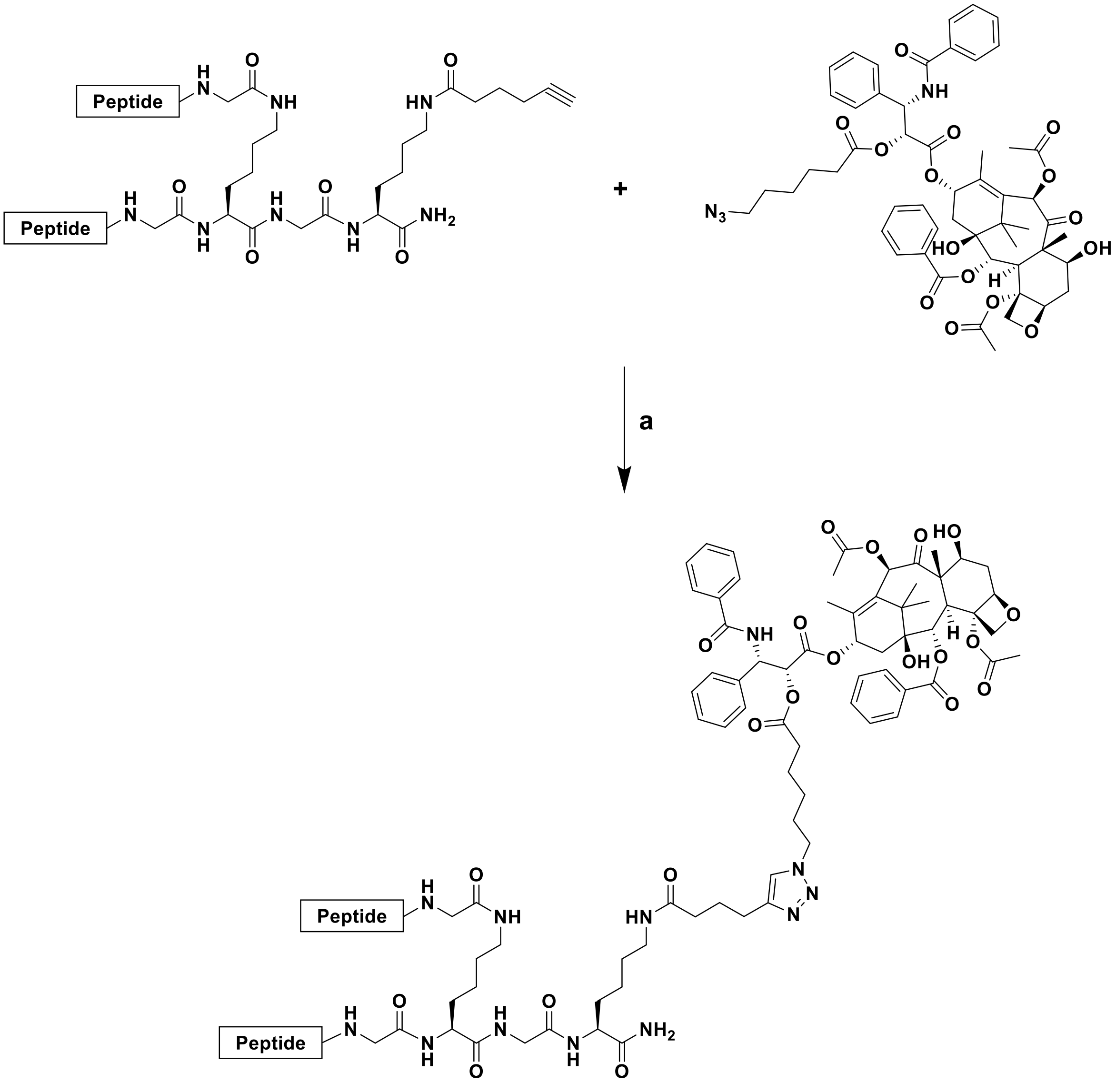

Figure S7. **Synthetic scheme for the synthesis of Targefrin-dimer-TAMRA** . Conditions: **(a)** *Targefrin-dimer-motif* crude, 1 equiv. of 5-TAMRA-Azide in 4 mL of 4:1 DMSO:water solution. Add 50 uL of CuSO4 1M and 50 uL of Sodium Ascorbate 1M. Mix at rt for 48 h.

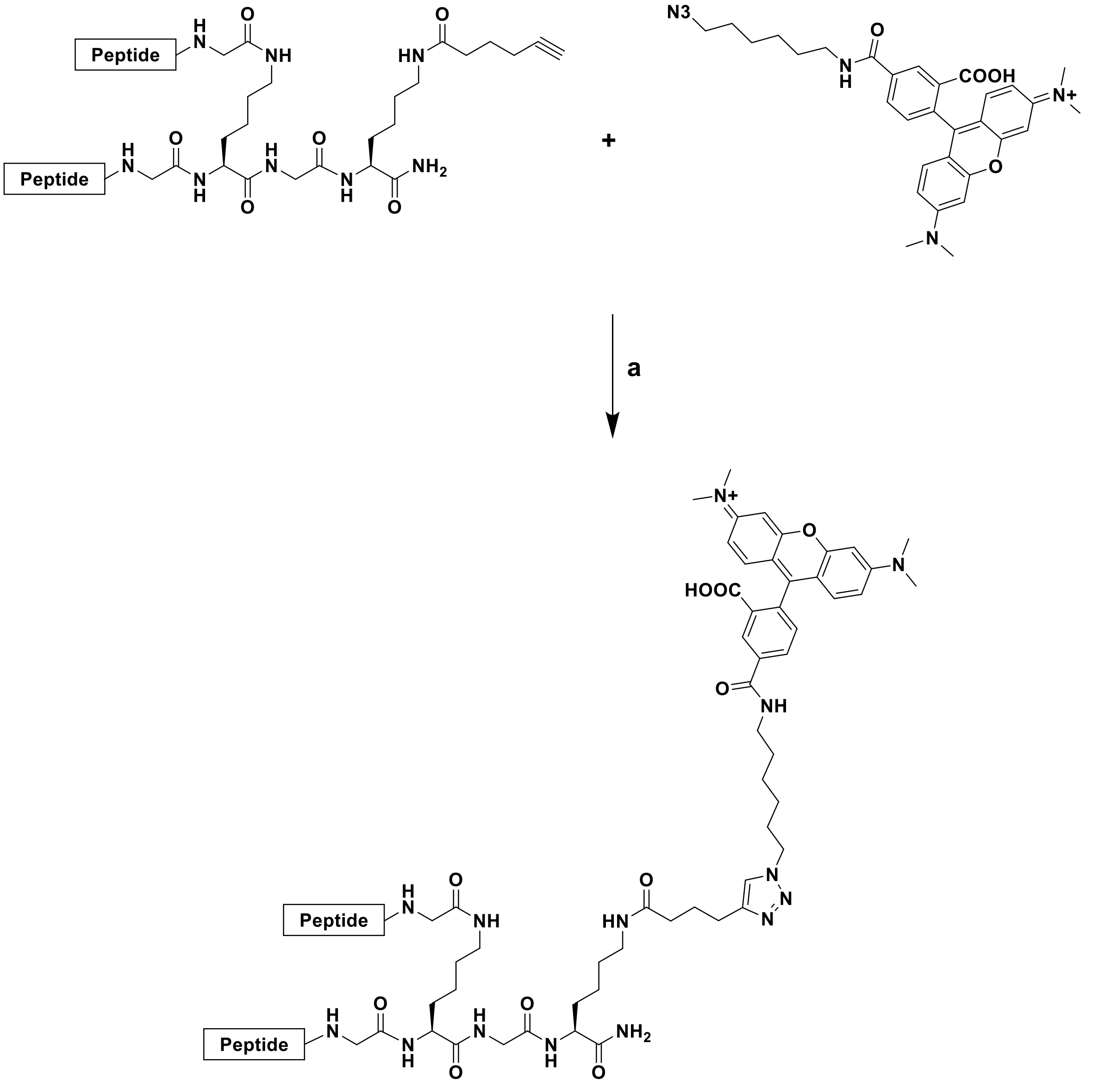

Figure S8. HPLC traces for tested compounds. Analytical run was accomplished using Atlantis T3 3μm 4.6x150 mm (H_2O/ACN gradient from 20% to 100% in 45 min). All compounds displayed purity > 95%.

Targefrin

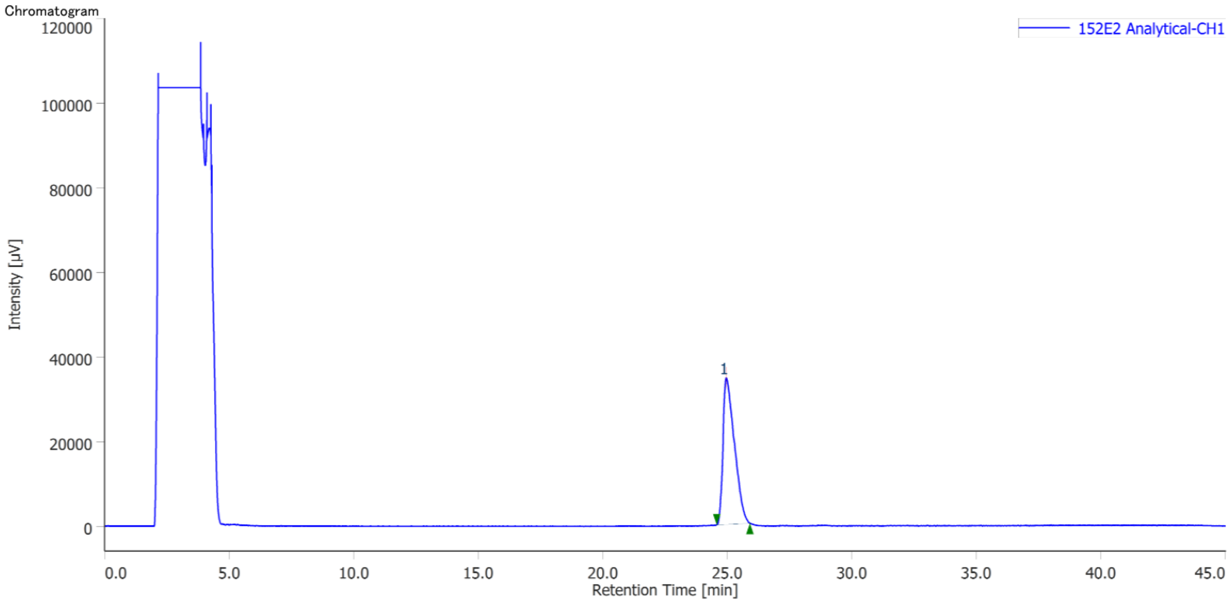

Peak Information

#	Peak Name	CH	tR [min]	Area [µV·sec]	Area%	Height [µV]	Height%	Resolution	Symmetry Factor
1	Unknown	1	24.952	1101207.900	100.000	34626	100.000	N/A	1.854

Targefrin-dimer

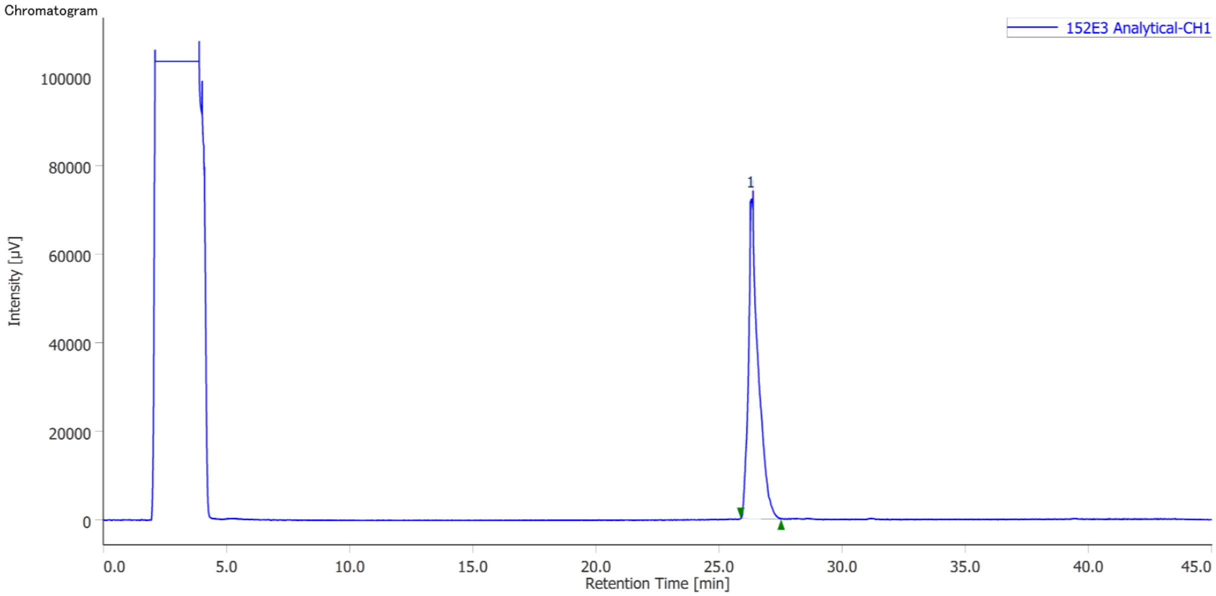

Peak Information

#	Peak Name	CH	tR [min]	Area [µV·sec]	Area%	Height [µV]	Height%	Resolution	Symmetry Factor
1	Unknown	1	26.373	1990309.522	100.000	73944	100.000	N/A	1.407

Targefrin-PTX

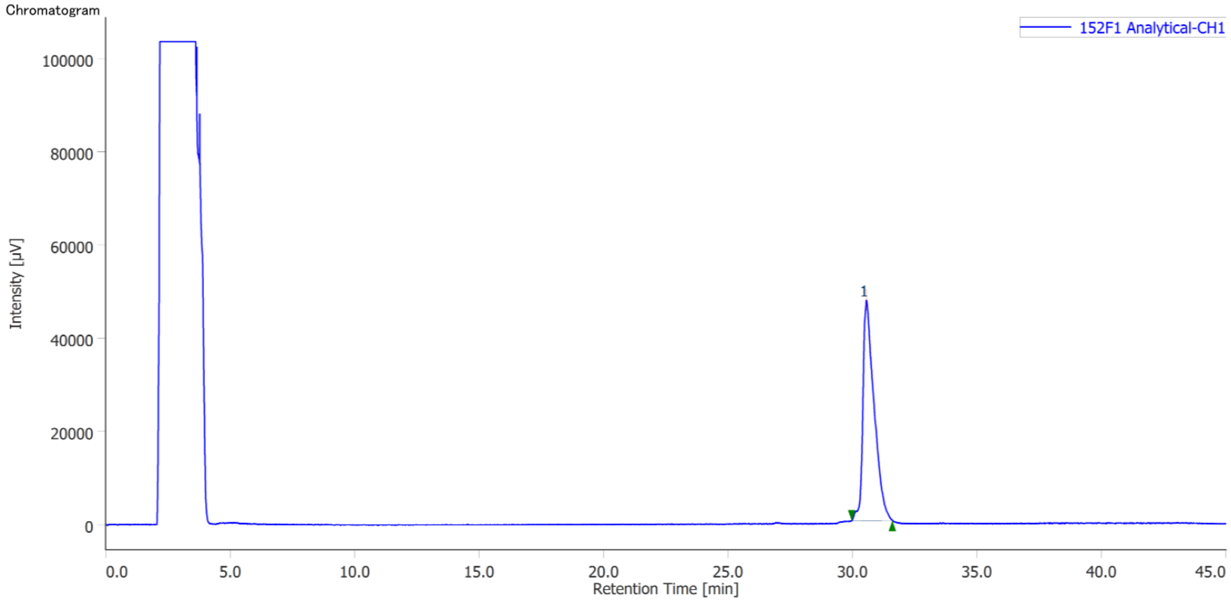

Peak Information

#	Peak Name	CH	tR [min]	Area [µV·sec]	Area%	Height [µV]	Height%	Resolution	Symmetry Factor
1	Unknown	1	30.550	1473735.400	100.000	47320	100.000	N/A	1.668

Targefrin-dimer-PTX

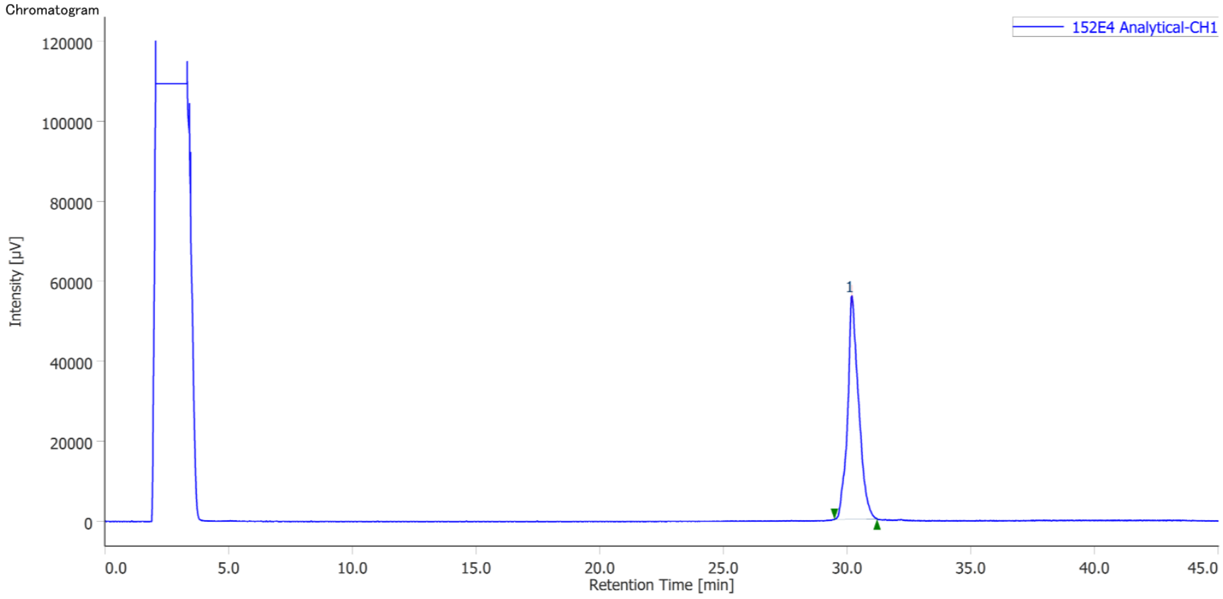

Peak Information

#	Peak Name	CH	tR [min]	Area [µV·sec]	Area%	Height [µV]	Height%	Resolution	Symmetry Factor
1	Unknown	1	30.187	1735509.200	100.000	55885	100.000	N/A	1.209

Figure S9. **Pharmacokinetics studies**. Preliminary pharmacokinetic (PK) studies with Targefrin-dimer. The agent has been injected IV via the tail vein at a concentration of 50 mg/Kg in a formulation of 80% PBS, 10% Tween 80, and 10% Ethanol. Note that is formulation resulted in a clear solution containing 20mg/ml of targefrin-dimer. C_{max} ~ 650 ng/mL after 2 hours from the injection. Estimated $t_{1/2}$ ~ 15 hr.

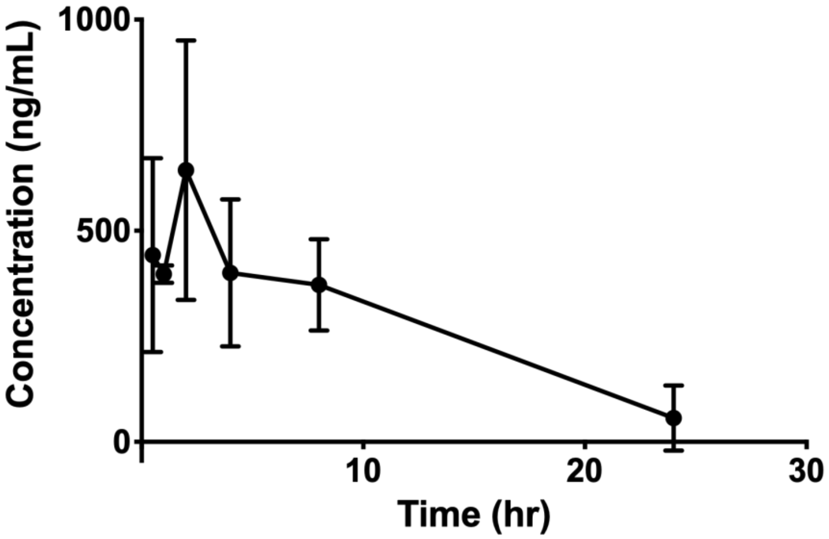

Figure S10. **Duplicate experiments reporting uncropped western blots that generated the data in Figures 4, 5 and 8.**

Figure 4. Expt 1

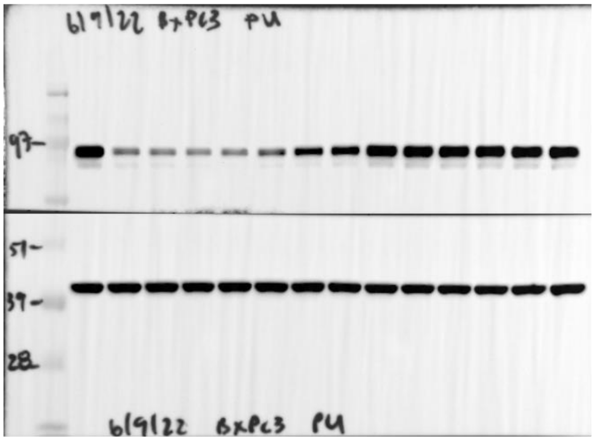

Figure 4. Expt 2

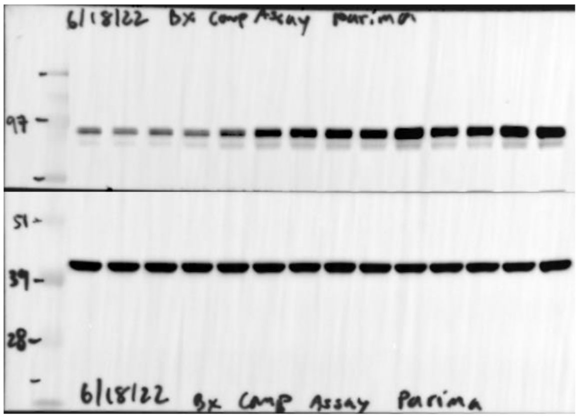

Figure 5. BxPC3 Expt 1

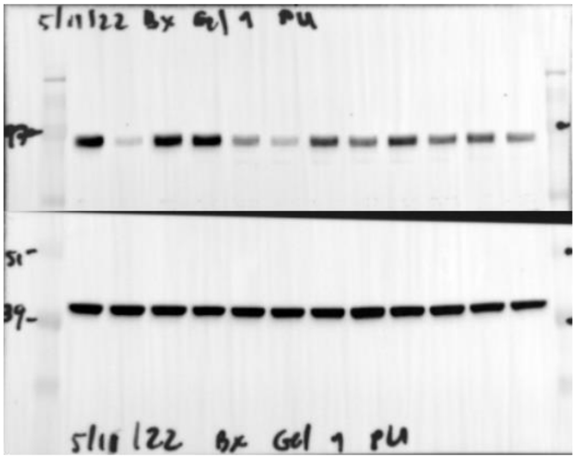

Figure 5. BxPC3 Expt 2

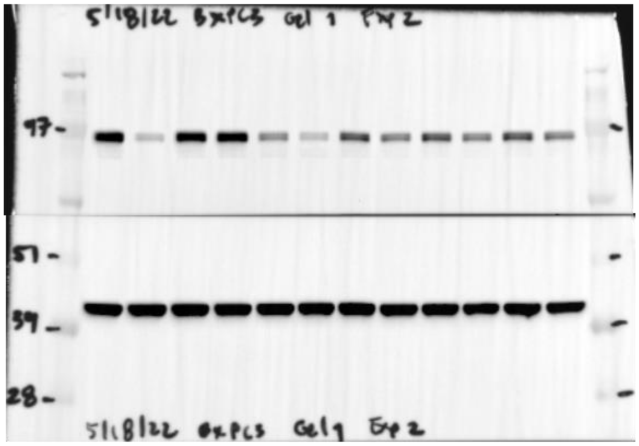

Figure 5. PANC-1 Expt 1

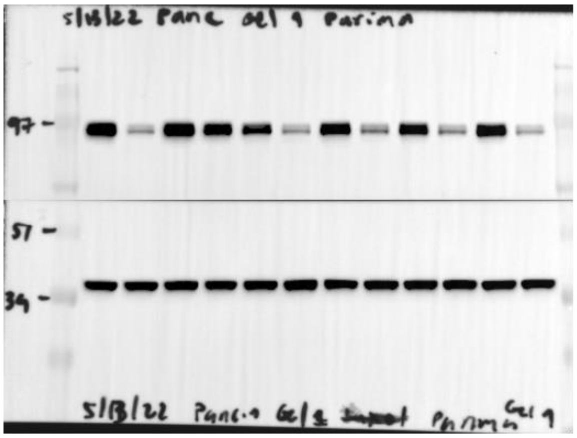

Figure 5. PANC-1 Expt 2

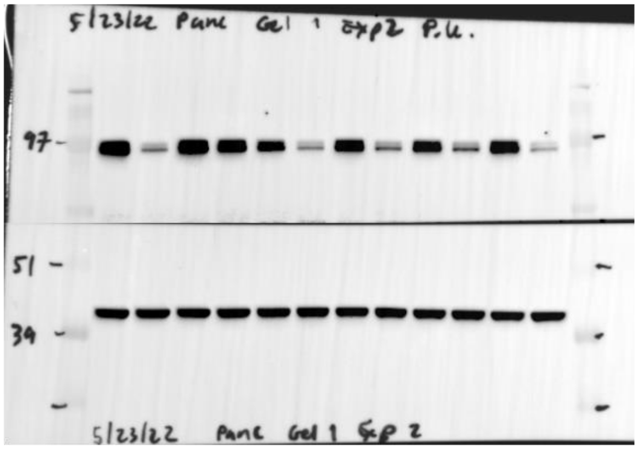

Figure 5. MIA PaCa-2 Expt 1

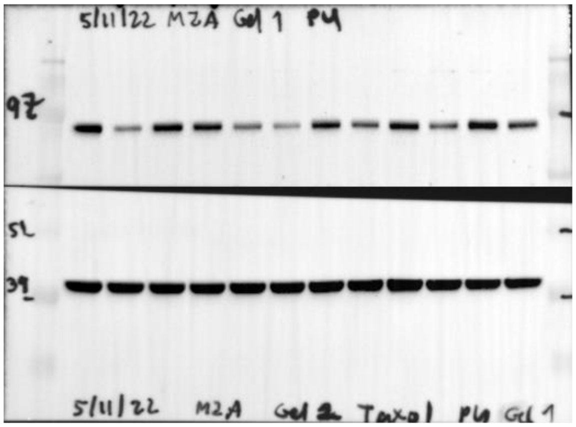

Figure 5. MIA PaCa-2 Expt 2

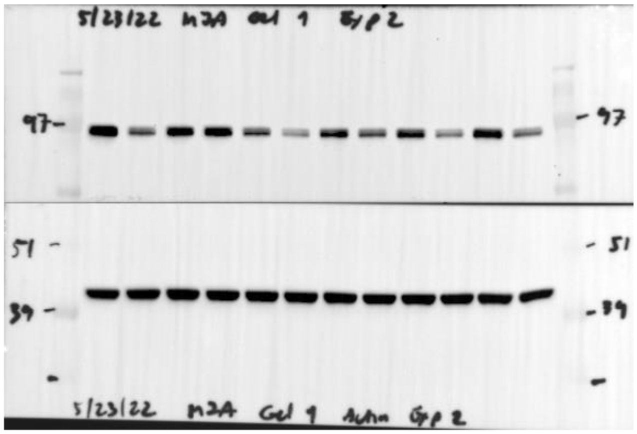

Figure 8. BxPC3 Expt 1

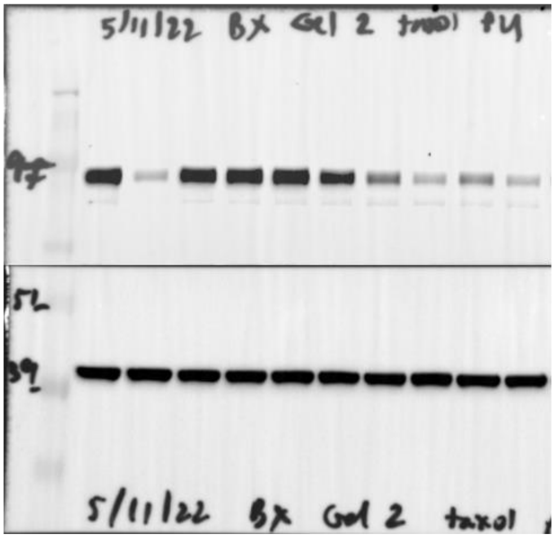

Figure 8. BxPC3 Expt 2

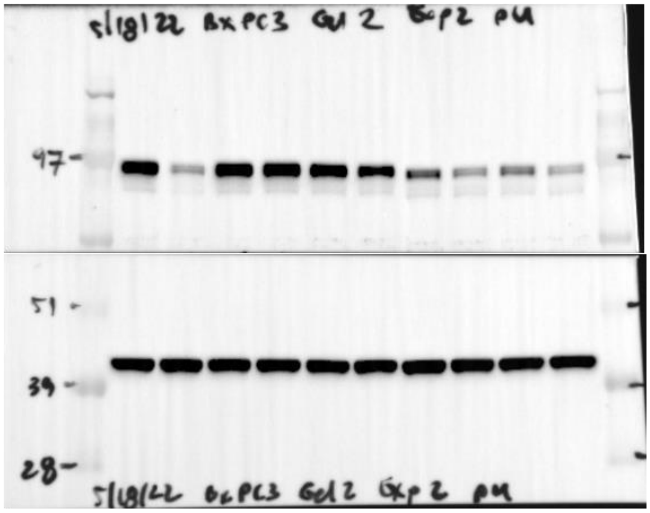

Figure 8. PANC-1 Expt 1

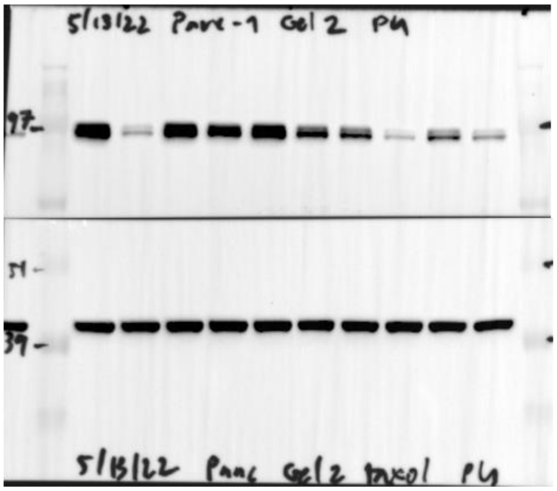

Figure 8. PANC-1 Expt 2

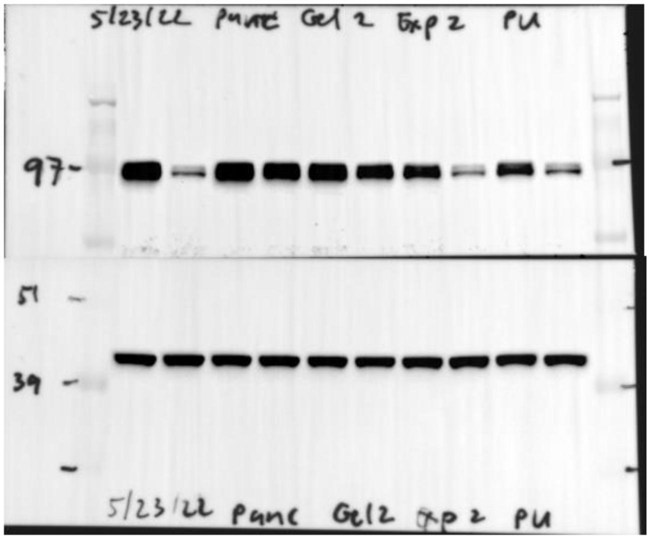

Figure 8. MIA PaCa-2 Expt 1

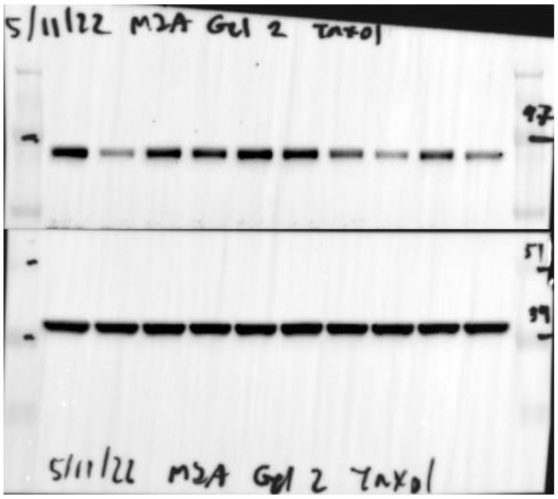

Figure 8. MIA PaCa-2 Expt 2

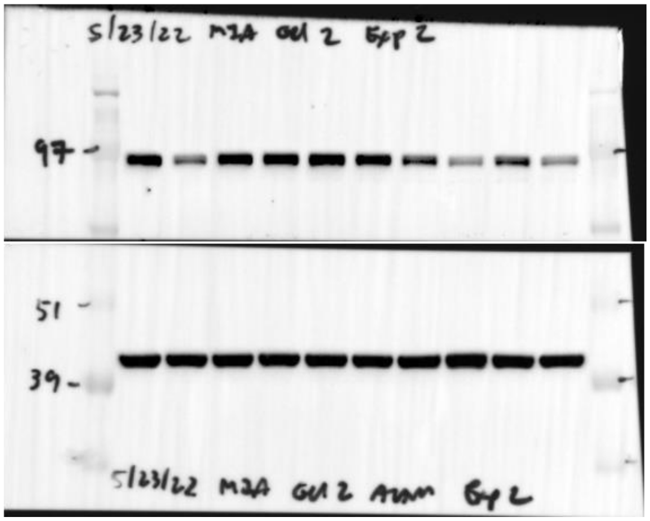

Figure S11. **Cell migration assay of BxPC3 from Figure 9 at 12 h. A**) Cell migration assay of BxPC3 treated with 2 μg/mL ephrinA1-Fc and 10 μM targefrin or the indicated doses of targefrin-dimer. The yellow lines displayed initial scratches made at 0 h while the black lines displayed the location that the cells had migrated to after 12 h. **B**) Targefrin-dimer significantly inhibited cell migration at 12 h in a dose-dependent manner as shown by decreases in relative wound density. ***$p < 0.01$, ****$p < 0.0001$, as determined by a one-way analysis of variance using Dunnett's post-test analysis.

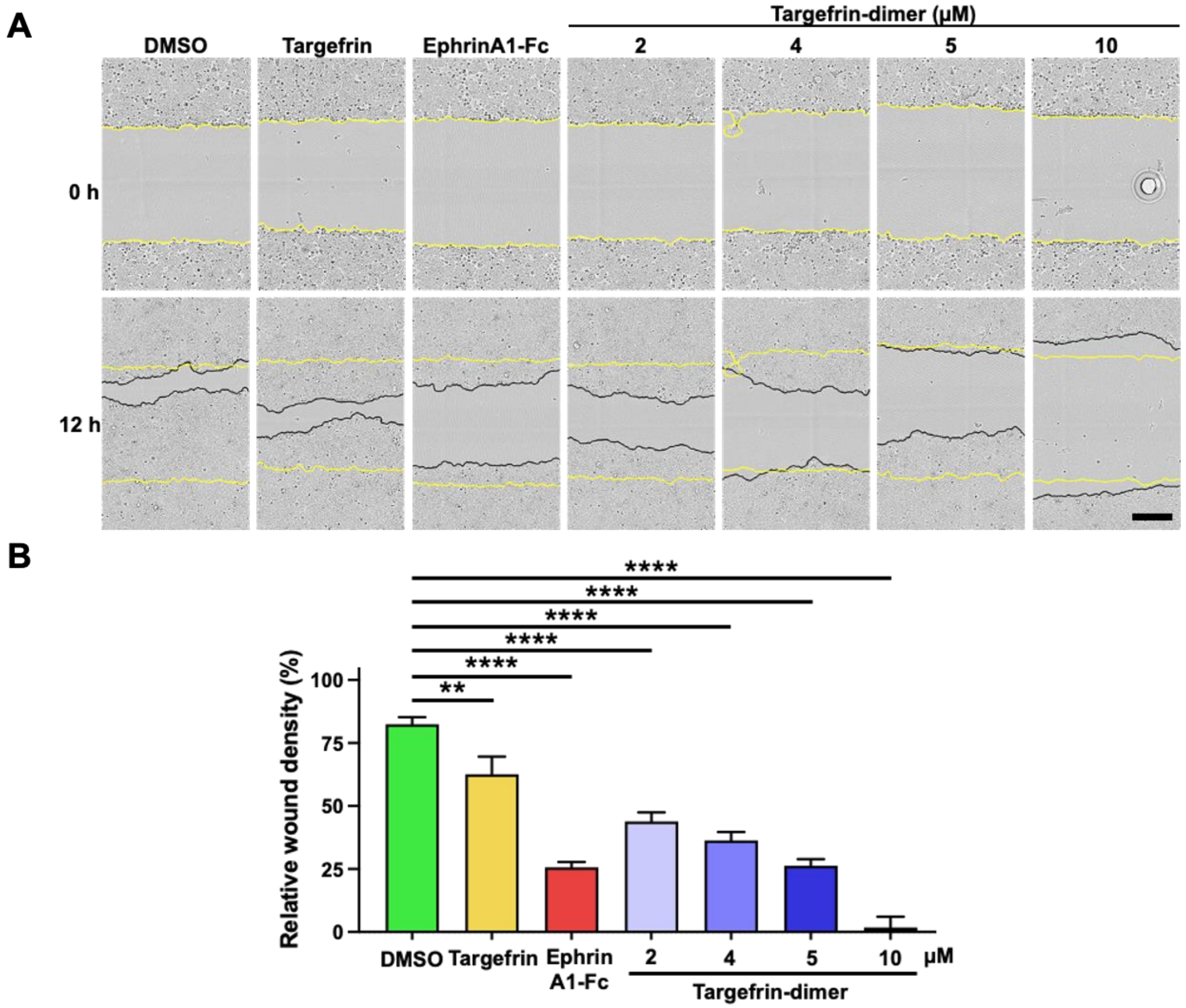

Figure S12. Cell viability assay of MIA PaCa-2 at 72 h. A) MIA PaCa-2 cells were treated with 1 µg/mL ephrinA1-Fc, different doses of Targefrin or Targefrin-dimer for 72 h. Percent confluency was monitored with the IncuCyte S3 live-cell analysis system and percent cell viability was calculated by normalizing the confluency of the treatments to that of the DMSO control. Percent cell viability was not significantly affected across all the treatments, as determined by a two-way analysis of variance using Bonferroni post-test analysis. **B**) Time-response curves for the percent confluency of MIA PaCa-2 cells after the indicated treatments.

A

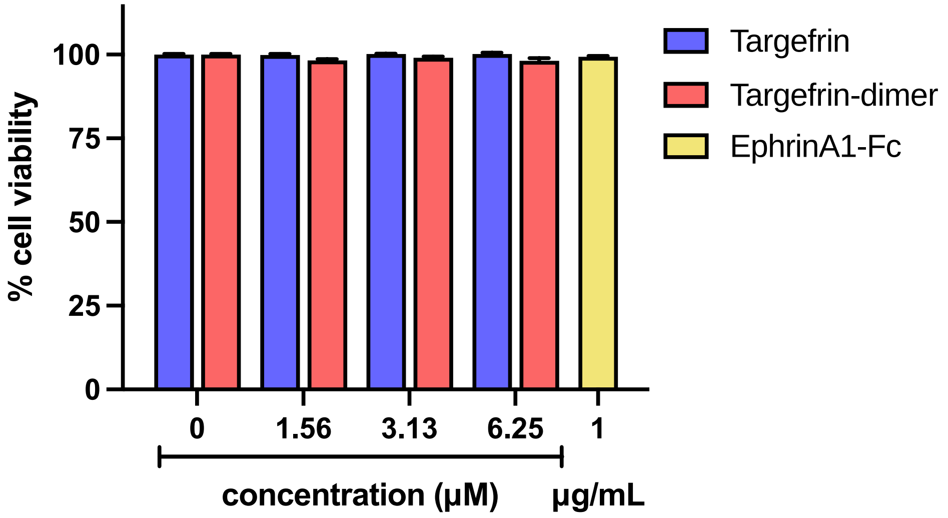

B

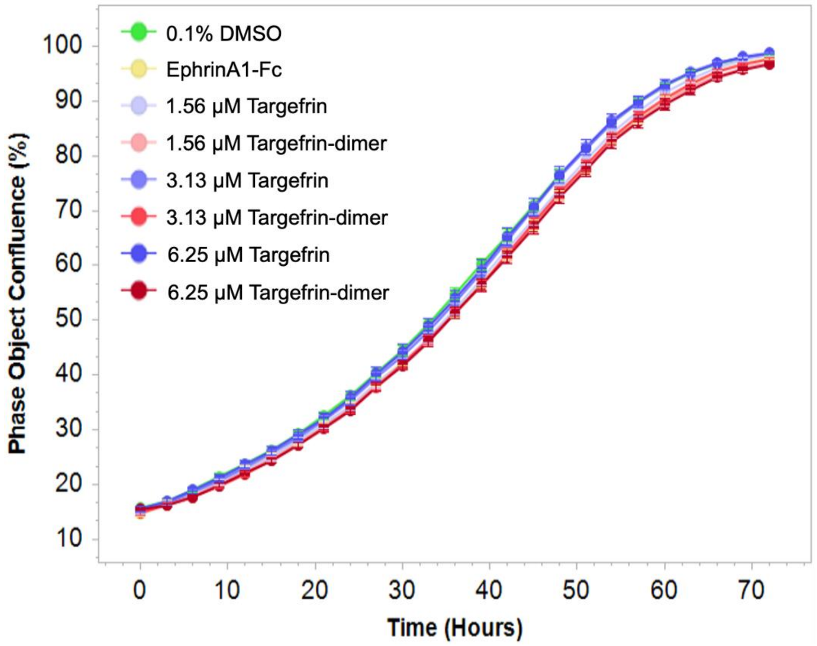

Figure S13. Blood chemistry analysis for mice treated with Targefrin-dimer. 5 Balb/c mice were treated with Targefrin-dimer 20 mg/mL (80% PBS, 10% Tween 80, 10% Ethanol) to obtain 50 mg/kg doses. After 24 hr mice were sacrificed and full chemistry panel analysis was conducted as listed below.

Analyte Name		Units	Reference Ranges	153E3/ IV #24	153E3/ IV #25	153E3/ IV #26	153E3/ IV #27	153E3/ IV #28
Albumin	ALB	g/dL	2.5 - 4.8	3.1	3.6	3.7	4.1	3.0
Alkaline Phosphatase	ALP	U/L	62 - 209	95	107	102	105	76
Alanine Transaminase	ALT	U/L	28 - 132	182	88	64	50	244
Amylase	AMY	U/L	1691 - 3615	927	979	938	828	830
Bilirubin, total	TBIL	mg/dL	0.1 - 0.9	0.3	0.3	0.3	0.3	0.3
Blood Urea Nitrogen	BUN	mg/dL	18 - 29	15	14	15	14	21
Calcium	CA	mg/dL	5.9 - 9.4	9.4	9.6	9.9	10.2	9.4
Phosphorus	PHOS	mg/dL	6.1 - 10.1	7.0	7.0	6.3	5.5	9.2
Creatinine	CRE	mg/dL	0.2 - 0.8	0.3	0.2	0.2	0.5	0.3
Glucose	GLU	mg/dL	90 - 192	143	156	168	182	155
Sodium	NA+	mmol/L	126 - 182	158	156	155	159	156
Potassium	K+	mmol/L	4.7 - 6.4	6.5	6.8	7.0	7.1	7.2
Total Protein	TP	g/dL	3.6 - 6.6	5.0	5.2	5.2	5.7	4.9
Globulin, calculated	GLOB	g/dL	N/A	1.8	1.6	1.6	1.7	1.8

Figure S14: Repeated doses toxicity studies with Targefrin-dimer-PTX versus PTX alone. Balc/c mice received equimolar doses of PTX or targefrin-dimer-PTX daily (IV), and body weight were measured daily. FD = found dead. By day 5 all 3 mice in the PTX treated group were found dead. Mice treated with targefrin-dimer-PTX were lethargic after the first doses but recovered. No signs of toxicity were noted in the mice treated with targefrin-dimer.

Mouse ID	Treatment	Body weight (g)				
	50 mg/Kg	Monday	Tuesday	Wednesday	Thursday	Friday
6	Targefrin-Dimer-PTX	26.17	25.33	25.71	25.82	26.28
7	Targefrin-Dimer-PTX	25.63	24.98	25.14	24.21	24.71
8	Targefrin-Dimer-PTX	26.17	25.33	26.08	25.71	25.1
	8 mg/Kg					
9	PTX	23.77	23.22 (FD)	.-----.	.-----.	.-----.
10	PTX	23.92	23.63 (FD)	.-----.	.-----.	.-----.
11	PTX	23.61	21.82	21.85	21.95	22.11 (FD)
	50 mg/Kg					
12	Targefrin-dimer	24.48	24.14	23.83	23.21	22.94
13	Targefrin-dimer	22.63	22.25	22.71	22.58	22.11
14	Targefrin-dimer	24.07	23.34	23.55	23.61	23.98
